# Long‐Term Impact of Guselkumab on Skin, Sexuality, and Perceived Stigmatization in Patients With Psoriasis in Routine Clinical Practice: Week 76 Effectiveness and Safety Results From the Prospective German Multicenter G‐EPOSS Study

**DOI:** 10.1111/1346-8138.17866

**Published:** 2025-08-09

**Authors:** Sascha Gerdes, Peter Weisenseel, Durdana Groß, Rolf Ostendorf, Sebastian Zimmer, Adriana Otto, Friedemann J. H. Taut, Judita Makuc, Simmy Jacobsen, Nina Trenkler, Juliane Behrens, Dariusch Mortazawi

**Affiliations:** ^1^ Psoriasis Center Kiel, Department of Dermatology University Medical Center Schleswig‐Holstein, Campus Kiel Kiel Germany; ^2^ Dermatologikum Hamburg Hamburg Germany; ^3^ Dermatology Practice Dr. Med. Durdana Groß Potsdam Germany; ^4^ ZENTderma Mönchengladbach Germany; ^5^ Dermatology Practice Hautmedizin Saar Merzig Germany; ^6^ MediCorium Oberursel Germany; ^7^ Taut Science and Service GmbH Konstanz Germany; ^8^ Janssen‐Cilag GmbH, a Johnson & Johnson Company Neuss Germany; ^9^ Dermatology Practice Dariusch Mortazawi Remscheid Germany

**Keywords:** guselkumab, perceived stigmatization, psoriasis, quality of life, real‐world evidence, sexuality

## Abstract

**Background:**

G‐EPOSS is a prospective, non‐interventional, German multicenter study evaluating the effects of guselkumab, an interleukin‐23 inhibitor, in patients with moderate‐to‐severe plaque psoriasis in real‐world clinical practice.

**Objective:**

To evaluate the effectiveness of guselkumab in psoriasis and its impact on quality of life (QoL), sexual impairment, and perceived stigmatization.

**Methods:**

Adult patients received guselkumab according to routine clinical practice. Primary endpoint (Psoriasis Area and Severity Index [PASI] ≤ 3 at week [W]28) data are published. Secondary endpoints over 76 weeks include PASI, Nail Psoriasis Severity Index (NAPSI), anogenital Physician's Global Assessment (aPGA), Dermatology Life Quality Index (DLQI), Relationship and Sexuality Scale (RSS), Perceived Stigmatization Questionnaire (PSQ), Patient Benefit Index (PBI), and drug survival assessments. Outcomes were evaluated in the overall population and subgroups defined by body mass index (BMI), age, disease duration, sex, anogenital psoriasis, depression, and super‐response to guselkumab (PASI = 0 at W20 and W28).

**Results:**

A total of 295 patients were included in these analyses. Baseline mean disease duration, PASI, and aPGA scores were 17.4 years, 15.3, and 2.7, respectively. In total, 26.4% of patients had received prior biologic therapy. At W76, 87.5% of patients achieved PASI ≤ 3, and 47.3% achieved PASI = 0; response rates were higher with shorter disease duration. Overall, 18.3% of patients were super‐responders. Among patients with NAPSI ≥ 1 or aPGA ≥ 1 at baseline, NAPSI = 0 and aPGA = 0 were achieved by 52.2% and 75.8% of patients at W76, respectively. A high aPGA = 0 response rate was observed in all BMI subgroups. Improvements were observed through W76 across individual items of the DLQI, RSS, and PSQ and across all subgroups evaluated. A shorter disease duration was associated with additional benefit for some outcomes. At W76, PBI > 3 was documented for 88.1% of patients, and the probability of drug survival was 88.7%. No new safety signals were detected.

**Conclusions:**

Guselkumab treatment provided sustained improvements over 76 weeks in psoriasis, sexual impairment, QoL, and perceived stigmatization, irrespective of BMI, age, disease duration, sex, presence of anogenital psoriasis, or depression.

## Graphical abstract

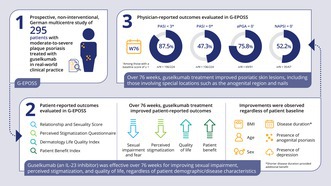

## 1 Introduction

Psoriasis is a chronic systemic inflammatory disease predominantly characterized by dry, scaly, itchy plaques on the skin [1, 2]. The disease burden extends beyond visible skin signs and symptoms and includes a complex interplay of physical, psychological, and social factors that significantly impact patients' lives [3, 4, 5, 6]. Patients with psoriasis have a higher risk of suffering from perceived stigmatization [7, 8, 9] and sexual impairment [10, 11] than healthy individuals. These conditions are becoming increasingly recognized as substantial contributors to the negative impact that psoriasis has on patients' overall health‐related quality of life (HRQoL) [12, 13]. Sexual impairment is experienced by more than half of all patients with psoriasis and is particularly associated with patients who have anogenital psoriasis [4, 10]. An even greater proportion of patients with psoriasis experience perceived stigmatization, which can result in or exacerbate depression [7, 8, 14]. Perceived stigmatization and depression can in turn have a negative impact on psoriasis severity [14, 15, 16]. Therefore, it is important that psoriasis is considered and treated using a holistic approach. However, while recent advancements in therapies have improved the physical signs and symptoms of psoriasis, their impact on patients' overall wellbeing remains incompletely understood. Further research is needed to explore this critical aspect of psoriasis and guide optimal treatment strategies.

Guselkumab is a fully human monoclonal antibody that binds to the p19 subunit of interleukin (IL)‐23 [17], with proven efficacy in patients with moderate‐to‐severe plaque psoriasis [18, 19, 20, 21]. The G‐EPOSS study aims to evaluate the long‐term effectiveness of guselkumab for improving psoriasis, HRQoL, sexual impairment, and perceived stigmatization outcomes in patients with moderate‐to‐severe plaque psoriasis in routine clinical practice. Primary endpoint findings were previously published [22]; here, we present key secondary endpoints from the final week (W)76 data cut, reporting outcomes across diverse patient subgroups.

## 2 Methods

### 2.1 Study Design

G‐EPOSS is a prospective, non‐interventional, multicenter study of adults with moderate‐to‐severe plaque psoriasis conducted in Germany [22]. Patients were enrolled between October 2019 and August 2021 at 44 sites and received guselkumab 100 mg at W0, W4, and every 8 weeks thereafter until study end (W76) according to local routine clinical practice and the summary of product characteristics guidelines. The study was conducted in accordance with the International Conference on Harmonization Good Clinical Practice guidelines, with approval from the respective study site research ethics committees. All patients provided written informed consent.

### 2.2 Patients

Enrolled patients were ≥ 18 years old, had a diagnosis of moderate‐to‐severe plaque psoriasis with baseline Psoriasis Area and Severity Index (PASI) > 3, and were candidates for systemic therapy. Patient ineligibility criteria are described in Appendix S1. Concomitant medications for psoriasis (excluding biologic therapy) were permitted according to routine care.

### 2.3 Endpoints and Assessments

The primary endpoint was the proportion of patients achieving PASI ≤ 3 at W28 [22]. Secondary endpoint assessments at W76 are described in Appendix S1 and included physician assessments: PASI, Nail Psoriasis Severity Index (NAPSI) [23], and anogenital Physician's Global Assessment (aPGA) [24, 25]; and patient‐reported assessments: Dermatology Life Quality Index (DLQI) [26, 27, 28], Patient Benefit Index (PBI) [29, 30], Relationship and Sexuality Scale (RSS) [31], and the Perceived Stigmatization Questionnaire (PSQ) [32, 33]. PBI scores range from 0 (treatment did not help at all) to 4 (treatment helped a lot) [29]. The RSS comprises 10 questions assessing sexual function, sexual frequency, and sexual fear during the previous 2 weeks [31]. The PSQ comprises 21 questions evaluating perceived stigmatization [32, 33]. The evaluable set population (ESP; patients who received ≥ 1 dose of guselkumab and had a PASI measurement after baseline) was analyzed. Analyses were also conducted in subgroups based on baseline sex, age, body mass index (BMI), disease duration, and presence/absence of anogenital psoriasis or depression (PSQ only), as well as super‐response to guselkumab (defined as PASI = 0 at W20 and W28) [34]. Safety was analyzed using the safety population (patients who received ≥ 1 guselkumab dose) at data cut‐off (17 March 2023) by evaluating adverse events (AEs), using terms defined by the Medical Dictionary for Regulatory Activities v24.0.

### 2.4 Statistical Analysis

Enrollment of approximately 300 patients was planned [22], with as‐observed analyses conducted for all endpoints. Mean scores and response rates are presented, and proportions of patients are reported for patient‐reported outcomes.

## 3 Results

### 3.1 Patients

Overall, 304 patients were included in the safety population, 295 patients were included in the ESP, and data were available for 225 patients at W76. There were 68 patient withdrawals prior to W76, including 13 (4.3%) because of AEs. Two patients did not have W76 data documented.

Baseline characteristics are shown in Table 1. Overall, 58.6% and 26.4% of patients had received prior systemic and biologic therapy, respectively, and 63.6% of the population had a disease duration of > 10 years.

**TABLE 1:** Patient baseline characteristics.

Characteristic	Total
Mean age, years (SD); *n =* 295	45.6 (14.5)
Sex, *n* (%); *n =* 295	
Male/female	172 (58.3)/123 (41.7)
Mean weight, kg (SD); *n =* 287	88.0 (19.5)
Mean BMI, kg/m^2^ (SD); *n =* 286	28.9 (6.1)
Mean height, cm (SD); *n =* 289	174.5 (9.8)
Mean age at first diagnosis, years (SD); *n =* 294	28.2 (15.6)
Mean duration of psoriasis, years (SD); *n =* 294	17.4 (13.4)
Mean duration of psoriasis, *n* (%); *n =* 294	
≤ 2 years	16 (5.4)
> 2–5 years	43 (14.6)
> 5–10 years	48 (16.3)
> 10 years	187 (63.6)
In a relationship, *n* (%); *n =* 267	
Yes, ≤ 5 years/> 5 years	48 (18.0)/164 (61.4)
No, single for ≤ 2 years/> 2 years	26 (9.7)/29 (10.9)
Mean PASI (SD); *n =* 295	15.3 (8.7)
Mean NAPSI (SD);^a^ *n =* 116	4.7 (3.3)
Mean aPGA (SD);^b^ *n =* 166	2.7 (0.9)
Mean DLQI (SD); *n =* 295	11.3 (6.6)
Comorbidities, *n* (%);^c^ *n =* 295	
Arterial hypertension	73 (24.7)
Psoriatic arthritis	75 (25.4)
Nicotine abuse	49 (16.6)
Obesity	39 (13.2)
Hypothyroidism	30 (9.9)
Depression	27 (9.2)
Diabetes mellitus	26 (8.8)
Dyslipidaemia	18 (6.1)
Metabolic syndrome	16 (5.4)
Prior psoriasis therapies,^d^, ^e^ *n* (%); *n* = 295	
Topical	121 (41.0)
Conventional systemic	173 (58.6)
Biologic	78 (26.4)
Number of prior biologics, *n* (%); *n* = 295	
0	217 (73.6)
1	43 (14.6)
≥ 2	35 (11.9)

Abbreviations: aPGA, anogenital Physician's Global Assessment; BMI, body mass index; DLQI, Dermatology Life Quality Index; NAPSI, Nail Psoriasis Severity Index; PASI, Psoriasis Area and Severity Index; SD, standard deviation.

^a^ Among patients with NAPSI ≥ 1.

^b^ Among patients with aPGA ≥ 1.

^c^ Comorbidities included in the table were present in ≥ 5% of patients.

^d^ Multiple responses were possible.

^e^ The proportions of patients receiving prior therapies are shown for the evaluable set population (*N =* 295), although prior therapy data were only available for 243 patients.

### 3.2 Clinical Effectiveness

**FIGURE 1:**
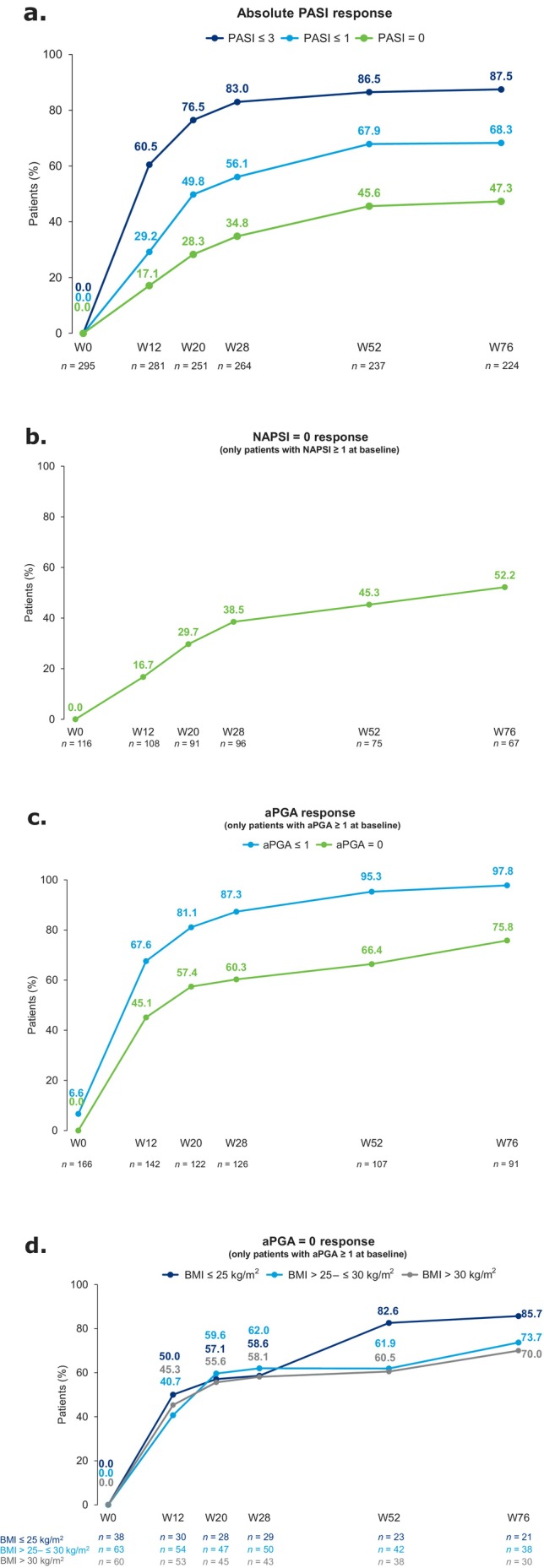
Proportions of patients achieving (a) PASI ≤ 3, PASI ≤ 1, and PASI = 0, (b) NAPSI = 0, (c) aPGA = 0 and aPGA ≤ 1, and (d) aPGA = 0 per BMI subgroup, from baseline to W76. Patients included in the NAPSI and aPGA response analyses had baseline NAPSI ≥ 1 and aPGA ≥ 1, respectively. aPGA, anogenital Physician's Global Assessment; BMI, body mass index; NAPSI, Nail Psoriasis Severity Index; PASI, Psoriasis Area and Severity Index; W, week.

Mean PASI decreased from 15.3 at baseline to 4.1 at W12, and to 1.2 by W76 (Figure S1). Meanwhile, absolute PASI response rates improved over time, with 87.5% (196/224) of patients achieving PASI ≤ 3, 68.3% (153/224) PASI ≤ 1, and 47.3% (106/224) PASI = 0 at W76 (Figure 1a). In total, 54/298 (18.3%) patients achieved super‐responder status. As the majority of the overall patient population had a disease duration of > 10 years, most super‐responders also had a mean disease duration of > 10 years (64.2%). However, super‐responders were less likely to have received prior biologics (18.5%) than the overall population. Of the super‐responders who had disease duration > 10 years (*n* = 34), only 23.5% had received prior biologics. PASI response rates were higher among super‐responders than the overall population, with 95.7% (44/46), 93.5% (43/46), and 82.6% (38/46) of super‐responders achieving PASI ≤ 3, PASI ≤ 1, and PASI = 0, respectively, at W76 (Figure S2).

PASI ≤ 3 and PASI = 0 response rates at W76 were similar among disease duration subgroups of ≤ 2 or > 2–5 years (respectively, 90.0% [9/10] and 93.1% [27/29] achieved PASI ≤ 3, 60.0% [6/10] and 72.4% [21/29] achieved PASI = 0; Figure S3). Patients with disease duration > 5–10 and > 10 years had PASI ≤ 3 response rates of 80.0% (32/40) and 88.2% (127/144), respectively, and substantially lower PASI = 0 response rates at W76 (42.5% [17/40] and 42.4% [61/144], respectively) than those with disease duration ≤ 5 years.

Among patients with NAPSI ≥ 1 at baseline, mean NAPSI improved from 4.7 at baseline to 1.3 at W76 (Figure S4), and 52.2% (35/67) achieved NAPSI = 0 at W76 (Figure 1b). Substantial improvements in anogenital psoriasis were observed (in patients with aPGA ≥ 1 at baseline); mean aPGA score decreased from 2.7 at baseline to 0.3 (0.1 among super‐responders) at W76 (Figures S5 and S6). Furthermore, 97.8% (89/91) of patients with aPGA ≥ 1 at baseline achieved aPGA ≤ 1 at W76 (only 6.6% [11/166] had aPGA ≤ 1 at baseline), and 75.8% (69/91) achieved aPGA = 0 (Figure 1c). Anogenital psoriasis was evaluated in BMI subgroups, and aPGA scores decreased substantially through W76 (to 0.1–0.4) for each subgroup (Figure S7). A high rate of aPGA = 0 response was observed across BMI subgroups; W76 aPGA = 0 response was numerically higher (85.7%) in the BMI ≤ 25 kg/m^2^ subgroup (Figure 1d).

### 3.3 Patient‐Reported Outcomes

#### 3.3.1 DLQI

**FIGURE 2:**
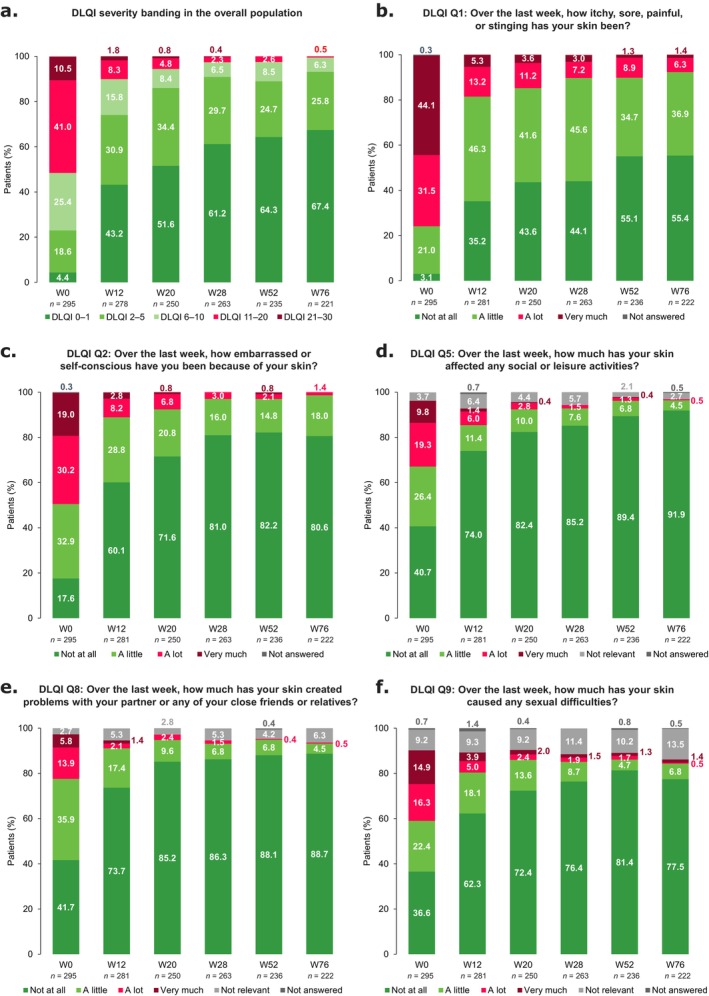
DLQI outcomes from baseline to W76. (a) DLQI severity banding in the overall population, and individual DLQI questions showing patient responses from baseline to W76 for (b) Q1, (c) Q2, (d) Q5, (e) Q8, and (f) Q9. Q1 reflects how much quality of life is affected by the skin itself, Q2 and Q8 are related to stigmatization, Q5 summarizes the impact of disease on social/leisure activities, and Q9 is related to sexual impairment. DLQI, Dermatology Life Quality Index; PASI, Psoriasis Area and Severity Index; Q, Question; W, week.

Patient HRQoL improved from baseline to W76; 67.4% (149/221) of patients achieved DLQI = 0/1 at W76 (4.4% [13/295] at baseline; Figure 2a), while mean DLQI decreased from 11.3 at baseline to 1.5 at W76 (Figure S8). Among patients with aPGA > 1 at baseline, those achieving aPGA ≤ 1 at W28 (*n* = 110) had lower mean DLQI through W76 than those with aPGA > 1 at W28 (Figure S9). Further subgroup analyses demonstrated that DLQI improved from baseline to W76 regardless of disease duration (Figure S10).

Responses to individual questions of the DLQI questionnaire also demonstrated improvements in HRQoL following guselkumab treatment (Figures 2b–2f and S11). At W76, 7.7% (17/222) of patients reported that their skin had been ‘a lot’/‘very much’ itchy, sore, painful, or stinging over the past 7 days (75.6% [223/295] at baseline; Question [Q]1; Figure 2b), and only 1.4% (3/222) reported that they felt ‘a lot’/‘very much’ embarrassed or self‐conscious because of their skin over the past 7 days (49.2% [145/295] at baseline; Q2; Figure 2c). The proportion of patients who reported that their skin did ‘not at all’ affect social/leisure activities (40.7% [120/295] at baseline, 91.9% [204/222] at W76; Q5; Figure 2d) or create problems with a partner, friends, or relatives (41.7% [123/295] at baseline, 88.7% [197/222] at W76; Q8; Figure 2e) increased at each visit. Furthermore, at W76, 1.8% (4/222) of patients reported that their skin condition had caused sexual difficulties ‘a lot’/‘very much’ over the past 7 days, compared with 31.2% (92/295) at baseline (Q9; Figure 2f).

#### 3.3.2 RSS

**FIGURE 3:**
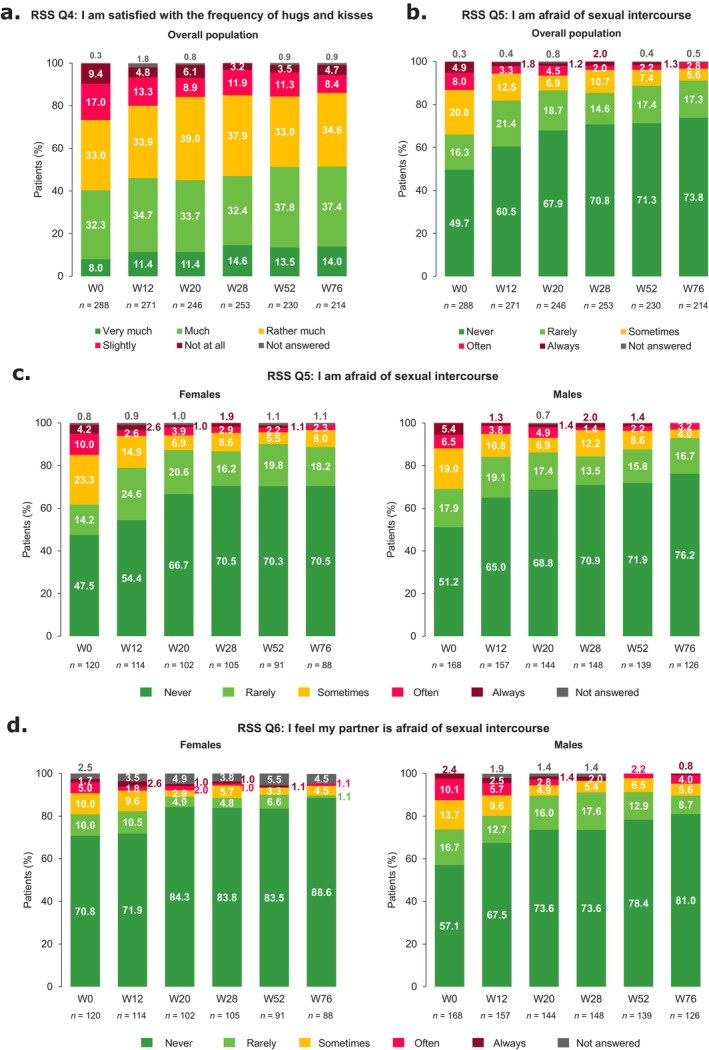
RSS Q4, Q5, and Q6 responses from baseline to W76. Individual RSS questions showing patient responses from baseline to W76 among the overall population for (a) Q4 and (b) Q5, and in subgroups defined by sex for (c) Q5 and (d) Q6. Q, Question; RSS, Relationship and Sexuality Scale; W, week.

**FIGURE 4:**
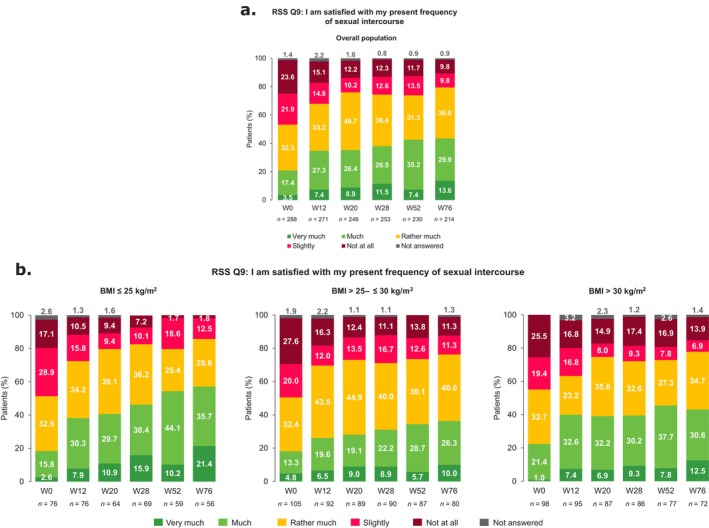
RSS Q9 responses from baseline to W76. RSS Q9 responses from baseline to W76 in the (a) overall population and (b) subgroups of patients defined by BMI. BMI, body mass index; Q, Question; RSS, Relationship and Sexuality Scale; W, week.

Sexual impairment improved from baseline to W76 (Figures 3a,b, 4a, and S12). Among patients not sexually active at baseline (*n* = 100), 29.0% (27/93) reported being sexually active by W12; this proportion remained relatively constant through W76 (35.1% [27/77]).

For individual RSS questions, 26.4% (76/288) of patients reported that they were ‘slightly’/‘not at all’ satisfied with their frequency of hugs and kisses at baseline, which decreased to 13.1% (28/214) at W76 (Q4; Figure 3a). Similar improvements were observed regardless of BMI (Figure S13a), sex (Figure S13b), and age (Figure S13c), although the 18–30 years group showed the largest improvement. Males and females with anogenital psoriasis at baseline had greater improvements in satisfaction through W76 than those without (Q4; Figure S13d).

Sexual fear also decreased over time; at baseline, 33.7% (97/288) of patients reported that they were ‘sometimes’, ‘often’, or ‘always’ afraid of sexual intercourse, compared with 8.4% (18/214) of patients at W76 (Q5; Figure 3b). Males were slightly less likely to report sexual fear than females, both at baseline and at W76 (Figure 3c). With guselkumab treatment, sexual fear decreased through W76 for both males and females with and without anogenital psoriasis (Figure S14a). Decrease in sexual fear from baseline to W76 with guselkumab treatment was also observed regardless of BMI or age (Figures S14b and S14c). Females were less likely than males to report that their partner was afraid of sexual intercourse (Q6; Figure 3d). Moreover, guselkumab treatment resulted in fewer patients reporting that their partners were afraid.

At baseline, 20.8% (60/288) of patients overall reported that they were ‘very much’/‘much’ satisfied with their frequency of sexual intercourse; this increased to 43.5% (93/214) at W76 (Q9; Figure 4a). Satisfaction increased through W76 regardless of BMI (Figure 4b), sex (Figure S15a), and age (Figure S15b), with the largest improvements in females and patients with BMI ≤ 25 kg/m^2^. Greater improvements in satisfaction through W76 were observed in males with versus without anogenital psoriasis, while a similar level of improvement was observed among females with and without anogenital psoriasis (Q9; Figure S15c).

#### 3.3.3 PSQ

**FIGURE 5:**
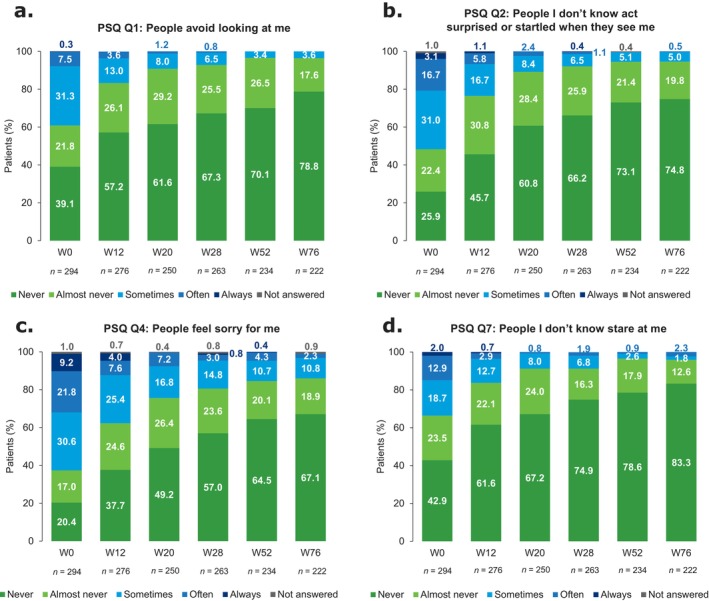
PSQ responses from baseline to W76 (overall population). Individual PSQ questions showing patient responses from baseline to W76 for (a) Q1, (b) Q2, (c) Q4, and (d) Q7. Responses for the selected questions shown here demonstrate some of the strongest improvements over time across all 21 PSQ questions; responses for all other questions are shown in Figure S16. PSQ, Perceived Stigmatization Questionnaire; Q, Question; W, week.

Following guselkumab treatment, patients reported a decrease in perceived stigmatization from baseline to W76 (Figures 5a–d and S16). At baseline, 39.1% (115/294) and 61.6% (181/294) of patients reported that people ‘sometimes’, ‘often’, or ‘always’ avoid looking at them (Q1; Figure 5a) and that people feel sorry for them (Q4; Figure 5c), respectively. This decreased to 3.6% (8/222) and 13.1% (29/222), respectively, at W76. Similar trends were observed for patients who reported that people they do not know act surprised or startled when they see them (Q2; Figure 5b), or that people they do not know stare at them (Q7; Figure 5d).

**FIGURE 6:**
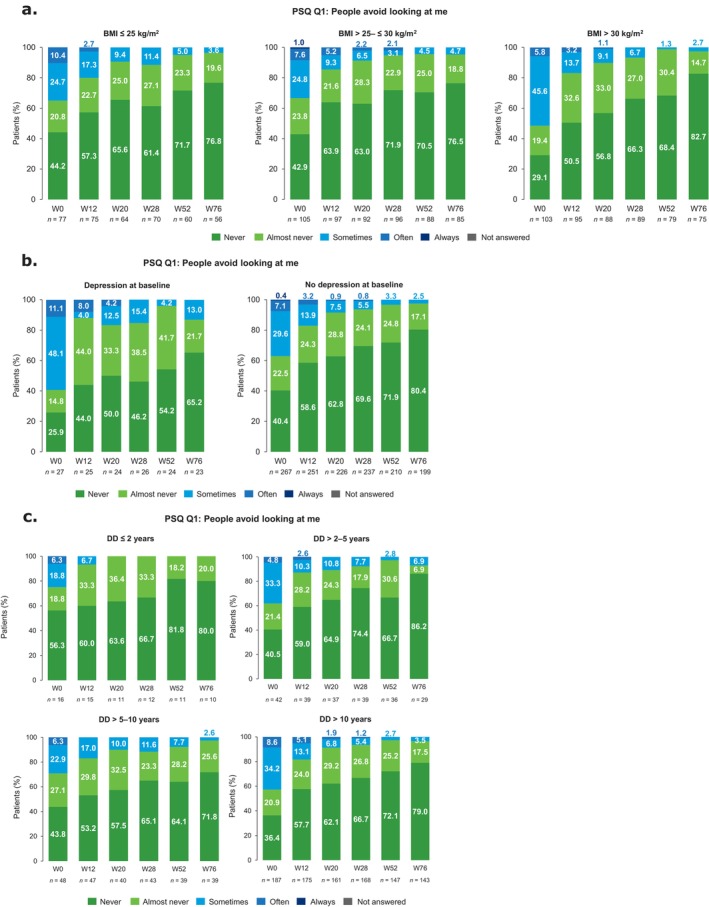
PSQ Q1 responses from baseline to W76 by different patient subgroups. PSQ Q1 responses from baseline to W76 in subgroups of patients defined by (a) BMI, (b) presence/absence of depression, and (c) disease duration. BMI, body mass index; DD, disease duration; PSQ, Perceived Stigmatization Questionnaire; Q, Question; W, week.

Perceived stigmatization decreased between W0 and W76 regardless of BMI (Figures 6a and S17) and presence of depression (Figures 6b and S18). Patients with depression were more likely than those without depression to report that people ‘sometimes’, ‘often’, or ‘always’ avoid looking at them at baseline (59.3% [16/27] vs. 37.1% [99/267], respectively) and at W76 (13.0% [3/23] vs. 2.5% [5/199], respectively; Figure 6b). Corresponding values for Q4 were 63.0% (17/27) and 61.4% (164/267) at baseline and 21.7% (5/23) and 12.1% (24/199) at W76 (Figure S18b). Similar findings were observed for PSQ Q2 and Q7 (Figure S18a,c). Further analyses showed that perceived stigmatization decreased through W76 for all disease duration subgroups (Figures 6c and S19). The ≤ 2 years subgroup experienced the least degree of perceived stigmatization.

#### 3.3.4 PBI

**FIGURE 7:**
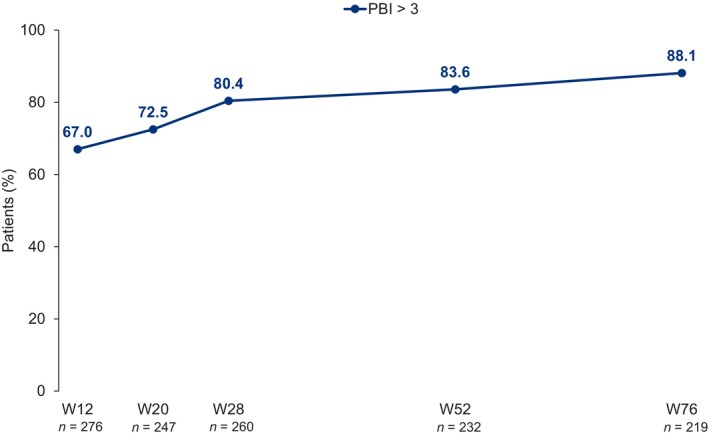
Proportion of patients reporting PBI > 3, from baseline to W76. PBI, Patient Benefit Index; W, week.

Most patients reported a relevant treatment benefit as early as W12 (the first time point assessed), with 67.0% of patients reporting PBI > 3 (Figure 7); this increased over time, reaching 88.1% (193/219) at W76.

#### 3.3.5 Drug Survival

**FIGURE 8:**
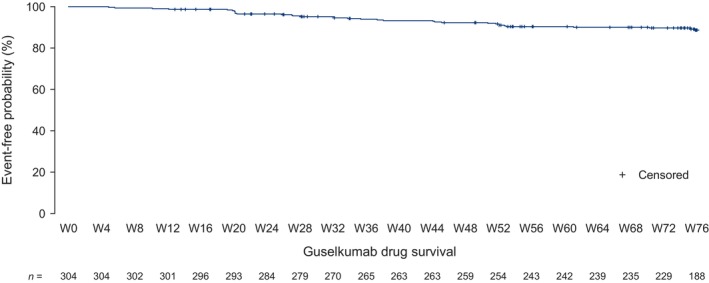
Probability of guselkumab drug survival over 76 weeks. Drug survival was assessed in the safety population by Kaplan–Meier analysis. Each censored event corresponds to a patient discontinuation, resulting from an adverse event, death, best interest of the patient, permanent guselkumab discontinuation, or regular termination of the study by the patient without continuation of treatment. The drop in number of patients at risk at W76 is a methodological artifact (i.e., most patients censored at W76 did not have a study visit at W76 but did have a visit within the allowed visit window) and is not due to medical reasons. W, week.

The probability of drug survival for guselkumab remained high throughout the study: at W76, the probability that patients were still on guselkumab treatment was 88.7% (Figure 8).

#### 3.3.6 Safety Outcomes

In total, 153 (50.3%) patients in the safety population (*N =* 304) experienced an AE, with the most common AEs comprising infections and infestations (Table 2). Overall, 30 (9.9%) patients experienced a drug‐related AE; the most common (≥ 2%) were administration‐site reactions, skin/subcutaneous tissue disorders, and infections/infestations (Table 2). Thirteen patients (4.3%) experienced 22 AEs that led to drug withdrawal. Two patients experienced four serious AEs (SAEs) that led to drug withdrawal. There were no drug‐related SAEs.

**TABLE 2:** Safety outcomes from baseline to week 76 (occurring in ≥ 2% of patients) by system organ class and preferred term (*N* = 304).

	Patients, *n* (%)	Events, *n*
Any AEs	153 (50.3)	361
Infections and infestations	72 (23.7)	105
COVID‐19	25 (8.2)	26
Nasopharyngitis	19 (6.3)	27
Skin and subcutaneous tissue disorders	47 (15.5)	56
Alopecia	6 (2.0)	6
Musculoskeletal and connective tissue disorders	30 (9.9)	35
Arthralgia	10 (3.3)	10
General disorders and administration‐site reactions	23 (7.6)	33
Fatigue	7 (2.3)	7
Nervous system disorders	16 (5.3)	25
Headache	8 (2.6)	11
Injury, poisoning, and procedural complications	15 (4.9)	18
Gastrointestinal disorders	13 (4.3)	16
Neoplasms: benign, malignant, and unspecified	12 (3.9)	13
Investigations	11 (3.6)	11
Surgical and medical procedures	10 (3.3)	10
Respiratory, thoracic, and mediastinal disorders	6 (2.0)	6
Any drug‐related AEs	30 (9.9)	63
General disorders and administration‐site reactions	14 (4.6)	22
Skin and subcutaneous tissue disorders	8 (2.6)	11
Infections and infestations	6 (2.0)	6
Any drug‐related AEs of at least moderate severity	11 (3.6)	28
General disorders and administration‐site reactions	6 (2.0)	11
Any AEs leading to drug withdrawal	13 (4.3)	22
General disorders and administration‐site reactions	8 (2.6)	9
Any SAEs	14 (4.6)	17
Any drug‐related SAEs	0 (0.0)	0
Any SAEs leading to drug withdrawal	2 (0.7)	4
Any SAEs leading to death	2 (0.7)	2

*Note:* SAEs leading to drug withdrawal were glioblastoma multiforme, metastatic lung cancer, cerebrovascular accident, and paraplegia (one event each). SAEs leading to death were malignant brain tumor (glioblastoma multiforme, which occurred during the safety follow‐up period) and cardiovascular failure.

Abbreviations: AE, adverse event; SAE, serious adverse event.

## 4 Discussion

The G‐EPOSS study provides extensive evaluation of guselkumab therapy in patients with moderate‐to‐severe psoriasis in routine clinical practice. To the authors' knowledge, G‐EPOSS is the first study to assess sexuality and perceived stigmatization in patients with psoriasis across a broad range of demographics and characteristics. Our findings further support the effectiveness of guselkumab in treating psoriasis, consistent with previous studies [19, 35, 36, 37, 38, 39], demonstrating steady improvements in mean PASI and PASI response rates through W76. G‐EPOSS findings further underscore the effectiveness of guselkumab in difficult‐to‐treat areas, namely, the anogenital region and nails.

As has been observed in previous real‐world studies [40], guselkumab was effective for psoriasis regardless of BMI, including in the anogenital region, where aPGA score improvement was similar across BMI subgroups. However, numerically higher rates of complete anogenital skin clearance at W76 were observed in patients with BMI ≤ 25 kg/m^2^ than in those with BMI > 25 kg/m^2^. Other studies have shown lower response rates for overweight or obese patients across biologic therapies, particularly in difficult‐to‐treat skin areas [40, 41].

We also evaluated the impact of disease duration on PASI response. Although subgroups with shorter disease duration were small, response rates for the highest treatment target (PASI = 0) were greatest among those with disease duration ≤ 5 years. Furthermore, PASI response rates at W76 were greatest among super‐responders (vs. the overall population), of whom 82.6% had completely clear skin, consistent with findings from the GUIDE trial [42].

Psoriasis also has a substantial impact on HRQoL [3, 4, 5, 6], sexuality [4, 10], and perceived stigmatization [7, 8, 14]. Using the DLQI, RSS, and PSQ, patients reported improvement or resolution in disease impact across broad subgroups of patients. Focusing on the RSS, with guselkumab treatment, fewer patients over time reported dissatisfaction with their frequency of hugs and kisses and sexual intercourse, as well as sexual fear, in the overall population and regardless of BMI or sex. Improvement in sexual impairment was reported across all age subgroups. At W76, all patients ≥ 60 years of age reported that they were ‘never’ or ‘rarely’ afraid of sexual intercourse.

Substantial improvements in RSS outcomes through W76 were reported by patients who suffered from anogenital psoriasis at baseline, likely linked to the decrease in mean aPGA score observed with guselkumab treatment. Greater improvements in sexual impairment were reported by patients who had anogenital psoriasis at baseline (who had more sexual dissatisfaction and fear at baseline) than by those who did not, consistent with the known impact of anogenital psoriasis on sexuality [43]. Further highlighting the substantial impact of anogenital psoriasis on sexual health and HRQoL [10, 22, 43, 44, 45, 46], our findings show that DLQI response was markedly better through W76 among those who achieved aPGA ≤ 1 at W28 than in those who did not. Our previous findings emphasized the importance of DLQI Q9 as a sentinel for anogenital involvement and sexual impairment [22].

Analyses of patients not sexually active at baseline also highlight the benefits of guselkumab for improving sexuality. Among these patients, 29.0% were engaging in sexual activity by W12.

Guselkumab rapidly decreased perceived stigmatization, and a low level of perceived stigmatization was maintained to W76. Moreover, improvement was observed irrespective of BMI at baseline, suggesting that the presence of visible psoriatic lesions, rather than elevated BMI, was the predominant cause of perceived stigmatization in obese psoriasis patients. Another study found that perceived stigmatization accompanying dermatological conditions was associated with increasing BMI; further studies are needed to address this topic [47].

Perceived stigmatization was also assessed in patients with and without depression, a common comorbidity in patients with psoriasis [48, 49, 50]. Previous findings indicate that common inflammatory processes are involved in the progression of both depression and psoriasis, and that a bidirectional relationship exists [49, 50]. Improvements in perceived stigmatization were reported regardless of the presence of depression, although patients with depression were more likely to report stigmatization at baseline and showed slower improvements over time to W76. Our findings highlight that depression can exacerbate the substantial impact that psoriasis has on HRQoL, and that treatment with guselkumab may be beneficial in patients with psoriasis and comorbid depression.

Perceived stigmatization decreased through W76 across all disease duration subgroups. Although patient numbers were small, the findings indicate that the initiation of guselkumab treatment within 2 years of diagnosis results in greater improvement or near‐complete resolution of stigmatization as early as W20. Mean DLQI also improved through W76 irrespective of disease duration; however, it was lowest at W76 in patients with disease duration ≤ 5 years. The impact of disease duration on psoriasis and patient‐reported outcomes highlights the importance of receiving guselkumab early in the disease course; although guselkumab is effective regardless of disease duration for patients in routine clinical practice, a better response is observed among those with short disease duration. These findings complement data from GUIDE, in which patients treated with guselkumab within ≤ 2 years from psoriasis symptom onset achieved better skin outcomes versus patients treated after > 2 years from symptom onset [51]. In GUIDE, patients with disease duration ≤ 2 years and those who were biologic naïve were more likely to achieve super‐responder status. The mean (SD) disease duration for super‐responders was 9.9 (12.4) years [51].

In G‐EPOSS, most patients (187/295 [63.6%]) had a disease duration of > 10 years at baseline, and our findings showed that patients with a disease duration of > 10 years can be super‐responders (64.2% of super‐responders had disease duration of > 10 years). Therefore, patients with longer disease duration can be super‐responders, although patients with a short disease duration have a higher probability of super‐response than patients with a long disease duration [51]. Notably, most of these super‐responders in G‐EPOSS with a disease duration of > 10 years were biologic naïve (76.5%), consistent with the overall G‐EPOSS population. This may be a hint that early treatment with guselkumab leads to the possibility of super‐response.

In addition to the substantial improvements observed across patient‐reported HRQoL measures, most patients reported PBI > 3 at W76, highlighting the strong benefit [29, 30] provided by guselkumab and indicating that patients' expectations of their therapy were met. Reflecting this, the probability of patients remaining on treatment at W76 was high (88.7%), consistent with previous analyses of guselkumab [19, 37, 52]. No new safety signals were observed; the overall dataset from this study affirms the positive benefit–risk profile of guselkumab in psoriasis management.

G‐EPOSS results provide valuable insights into sexual impairment and perceived stigmatization in patients with psoriasis in a real‐world setting. Guselkumab treatment over 76 weeks provided sustained improvements in outcomes related to psoriatic skin, sexual impairment, and HRQoL, and reduced perceived stigmatization, irrespective of BMI, age, disease duration, and depression. The findings emphasize the importance of involving the patient's view and considering sensitive topics not always openly communicated by patients. In summary, a holistic view of patients and their treatments is important for optimal care; G‐EPOSS supports findings that guselkumab not only addresses physical symptoms but also improves overall patient wellbeing.

## Ethics Statement

The study was conducted in accordance with the International Conference on Harmonization Good Clinical Practice guidelines and with approval from the respective research ethics committees. All patients provided written informed consent.

## Conflicts of Interest

Sascha Gerdes was an advisor for, received speakers' honoraria and/or grants from, and/or participated in clinical trials by AbbVie, Acelyrin Inc., Adimune Inc., Affibody AB, Akari Therapeutics Plc, Almirall Hermal, Amgen, Argenx BV, Boehringer Ingelheim, Bristol Myers Squibb, Celgene, Dermira, Eli Lilly, Galderma, Hexal AG, Incyte Inc., Janssen‐Cilag GmbH, Klinge Pharma, Kymab, LEO Pharma, Medac, Neubourg Skin Care GmbH, Novartis, Pfizer, Pierre Fabre, Principia Biopharma, Regeneron Pharmaceutical, Sandoz Biopharmaceuticals, Sanofi‐Aventis, and UCB Pharma. Peter Weisenseel was an advisor for, received speakers' honoraria and/or grants from, and/or participated in clinical trials by AbbVie, Almirall, Biogen, BMS, Celgene, Eli Lilly, Janssen‐Cilag GmbH, LEO Pharma, Medac, Novartis, Pfizer, and UCB Pharma. Durdana Groß was an advisor for, received speakers' honoraria and/or grants from, and/or participated in clinical trials by AbbVie, Almirall, Biogen, Bristol Myers Squibb, Celgene, Galderma, Janssen‐Cilag GmbH, LEO Pharma, MSD, Pfizer, Sanofi‐Aventis, and UCB Pharma. Rolf Ostendorf was an advisor for Amgen, Bristol Myers Squibb, Janssen‐Cilag GmbH, Novartis, and UCB Pharma, received speakers' honoraria from Almirall Hermal, Eli Lilly, LEO Pharma, and MedLight, received a travel grant from Janssen‐Cilag GmbH, and holds leadership or committee roles within Berufsverband der Deutschen Dermatologen and the European Academy of Dermatology and Venereology. Sebastian Zimmer was an advisor for, received speakers' honoraria and/or grants from, and/or participated in clinical trials by AbbVie, Almirall Hermal, Amgen, Boehringer Ingelheim, Bristol Myers Squibb, Celgene, Dermapharm, Eli Lilly, Galderma, Incyte Inc., Janssen‐Cilag GmbH, LEO Pharma, MSD, Novartis, Sanofi Genzyme, and UCB Pharma. Adriana Otto was an advisor for, received speakers' honoraria and/or grants from, and/or participated in clinical trials by AbbVie, Almirall, Bristol Myers Squibb, Dermapharm, Janssen‐Cilag GmbH, LEO Pharma, Merz, Sanofi, and Stallargenes. Friedemann J.H. Taut owns Taut Science and Service GmbH, a consultancy specialized in Clinical Development and Medical Affairs, and is receiving consultancy fees from Janssen‐Cilag GmbH, a Johnson & Johnson company. Judita Makuc, Simmy Jacobsen, Nina Trenkler, and Juliane Behrens are employees of Janssen‐Cilag GmbH, a Johnson & Johnson company. Dariusch Mortazawi was an advisor for, received speakers' honoraria and/or grants from, and/or participated in clinical trials by AbbVie, Almirall, Amgen, Bristol Myers Squibb, Eli Lilly, Janssen‐Cilag GmbH, LEO Pharma, LETI Pharma, Moberg Pharma, Novartis, Sandoz Biopharmaceuticals, Sanofi Genzyme, UCB Pharma, and Viatris.

## Supporting information


Data S1.

## Data Availability

The data that support the findings of this study are available from the corresponding author upon reasonable request. The data sharing policy of Janssen‐Cilag GmbH, a Johnson & Johnson company, is available at https://www.janssen.com/clinical‐trials/transparency. As noted on this site, requests for access to the study data can be submitted through the Yale Open Data Access Project site at http://yoda.yale.edu.
