# Supplementary material for: Long‐Term Impact of Guselkumab on Skin, Sexuality, and Perceived Stigmatization in Patients With Psoriasis in Routine Clinical Practice: Week 76 Effectiveness and Safety Results From the Prospective German Multicenter G‐EPOSS Study
Source: J Dermatol. 2025 Aug 9;52(9):1368–81. doi: 10.1111/1346-8138.17866 (PMC12411829; doi:10.1111/1346-8138.17866)
Supplement: Supplementary file 1 — Data S1. [file JDE-52-1368-s001.docx]

# Supplementary appendix

**Long-term impact of guselkumab on skin, sexuality, and perceived stigmatization in patients with psoriasis in routine clinical practice: week 76 effectiveness and safety results from the prospective German multicenter G-EPOSS study**

Supplement to S. Gerdes, P. Weisenseel, D. Groß, R. Ostendorf, S. Zimmer, A. Otto, F.J.H. Taut, J. Makuc, S. Jacobsen, N. Trenkler, J. Behrens, D. Mortazawi.

**Contents**

[Supplementary appendix 1](#_Toc172283710)

[Ineligibility criteria 2](#_Toc172283711)

[Reasons for study discontinuation 2](#_Toc172283712)

[Effectiveness and quality-of-life assessments 2](#_Toc172283713)

[Supplementary figures 4](#_Toc172283714)

[Clinical effectiveness outcomes 4](#_Toc172283715)

[Dermatology Life Quality Index outcomes 8](#_Toc172283716)

[Relationship and Sexuality Scale outcomes 11](#_Toc172283717)

[Perceived Stigmatization Questionnaire outcomes 20](#_Toc172283718)

[References 28](#_Toc172283719)

## Ineligibility criteria

Patients were not eligible for the study if they were pregnant or breastfeeding or had a clinically relevant active infection (e.g. tuberculosis).

## Reasons for study discontinuation

Patients discontinued from the study for the following reasons: initiation of a new concomitant prohibited therapy, withdrawal of consent, loss to follow-up, pregnancy, decision of the treating physician, or patient death.

## Effectiveness and quality-of-life assessments

Physician assessed:

- - Psoriasis Area and Severity Index (PASI), which evaluates the extent and severity of psoriasis and provides an overall score from 0 (no psoriasis) to 72 (severe form)^1^
  - Anogenital Physician’s Global Assessment (aPGA), which grades the patient’s anogenital psoriasis as clear (0), almost clear (1), mild (2), moderate (3), or severe (4)^2,3^
  - Nail Psoriasis Severity Index (NAPSI), used to document the presence of psoriasis in the nail matrix and nail bed in the four quadrants of a nail.^4^ NAPSI was evaluated in patients who had nail psoriasis at baseline. The best and worst nails at baseline were assessed throughout the study for each patient, providing a score ranging from 0 to 16 (0 to 8 for each of the two nails evaluated); higher scores represent more severe nail psoriasis

Patient reported:

- - Dermatology Life Quality Index (DLQI), comprising 10 questions that evaluate the impact of skin disease on the patient’s life during the previous week. Scores range from 0 to 30; a score of 0 or 1 indicates no effect of skin disease on quality of life. DLQI questions cover the following topics: symptoms and feelings, daily activities, leisure, work and school performance, personal relationships, and treatment^5-7^
  - Relationship and Sexuality Scale (RSS), comprising 10 questions that assess sexual function, sexual frequency, and sexual fear during the previous 2 weeks. Responses for each question are based on a four- or five-point Likert scale, providing a total score ranging from 10 to 46^8^
  - Perceived Stigmatization Questionnaire (PSQ), comprising 21 questions that assess perceived stigmatization and are answered on a five-point Likert scale, providing a score that reflects how the patient feels, ranging from 1 (never) to 5 (always). The question topics include ‘confused behavior and staring’ (eight items), the ‘absence of friendly behavior’ (eight reversed coded items), and ‘hostile behaviour’ (five items). Overall PSQ scores are calculated by adding the item responses and dividing the sum by the number of items; higher scores indicate higher levels of perceived stigmatization^9,10^
  - Patient Benefit Index (PBI), which comprises several aspects of patients’ expectations of their medical treatment, including clinical effectiveness, quality of life, and overall wellbeing. The total score ranges from 0 (treatment did not help at all) to 4 (treatment helped a lot).^11^ Previous findings in patients with psoriasis determined that PBI≥1 corresponds to a treatment providing a relevant benefit^12^

# Supplementary figures

## Clinical effectiveness outcomes

**Figure S1** Mean PASI from baseline to week 76


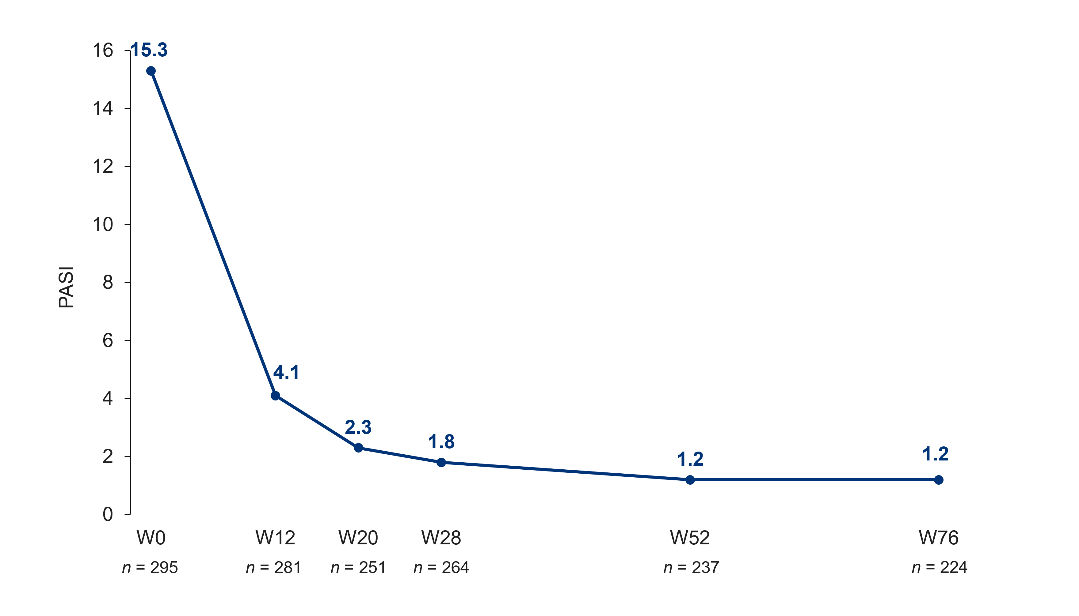


PASI, Psoriasis Area and Severity Index; W, week.

**Figure S2** Proportion of patients achieving PASI≤3, PASI≤1, and PASI=0 among super-responders, from baseline to week 76


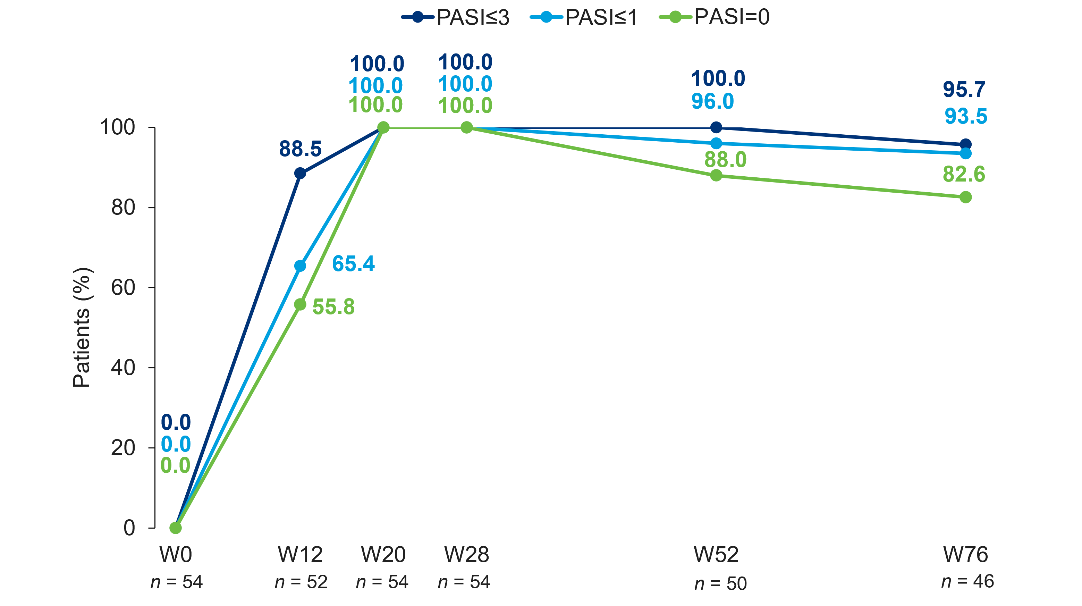


PASI, Psoriasis Area and Severity Index; W, week.

**Figure S3** Proportion of patients achieving PASI≤3 and PASI=0 among subgroups of patients defined by disease duration, from baseline to week 76


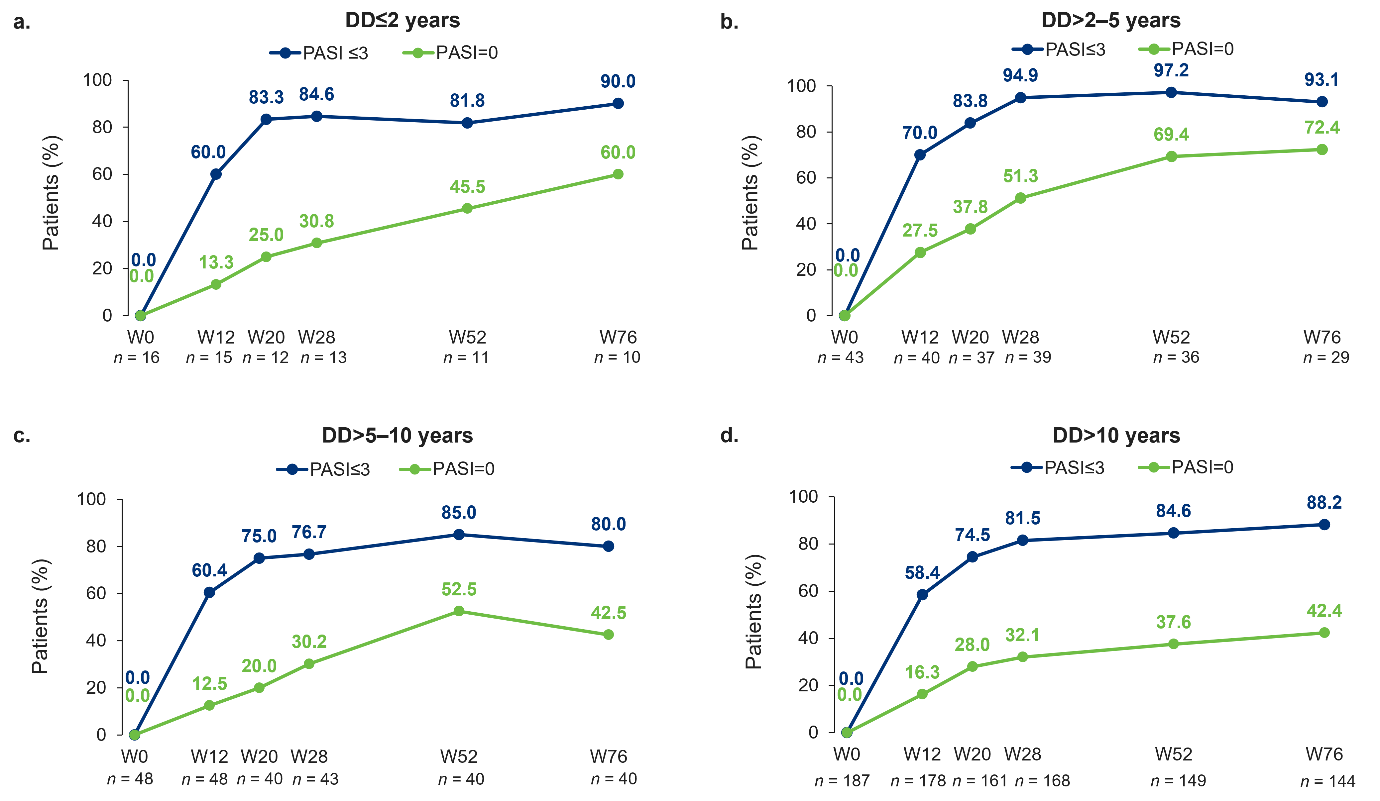


DD, disease duration; PASI, Psoriasis Area and Severity Index; W, week.

**Figure S4** Mean NAPSI from baseline to week 76


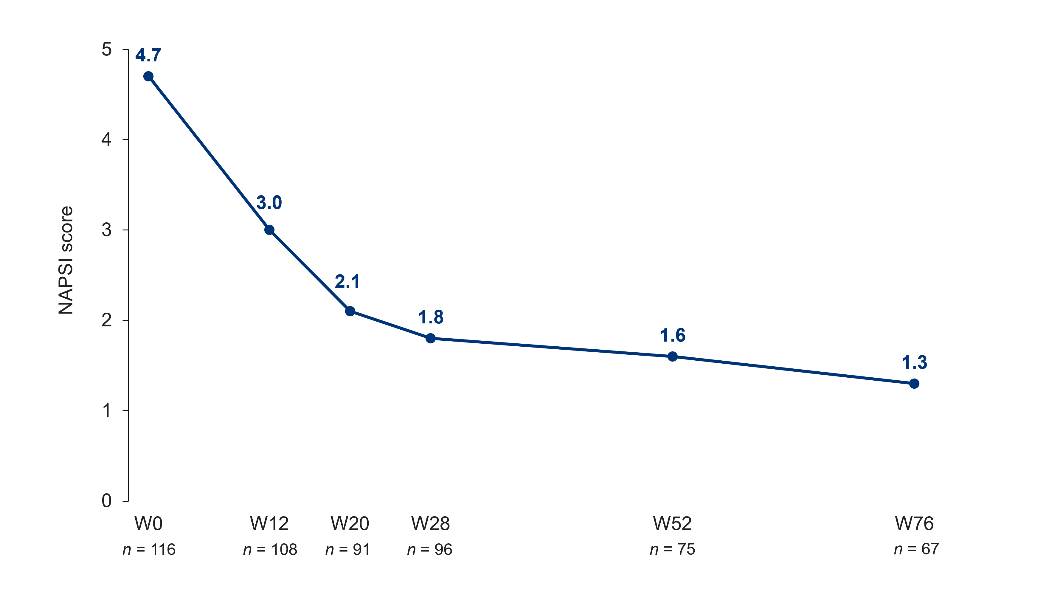


Analysis among patients with NAPSI≥1 at baseline.

NAPSI, Nail Psoriasis Severity Index; W, week.

**Figure S5** Mean aPGA score from baseline to week 76


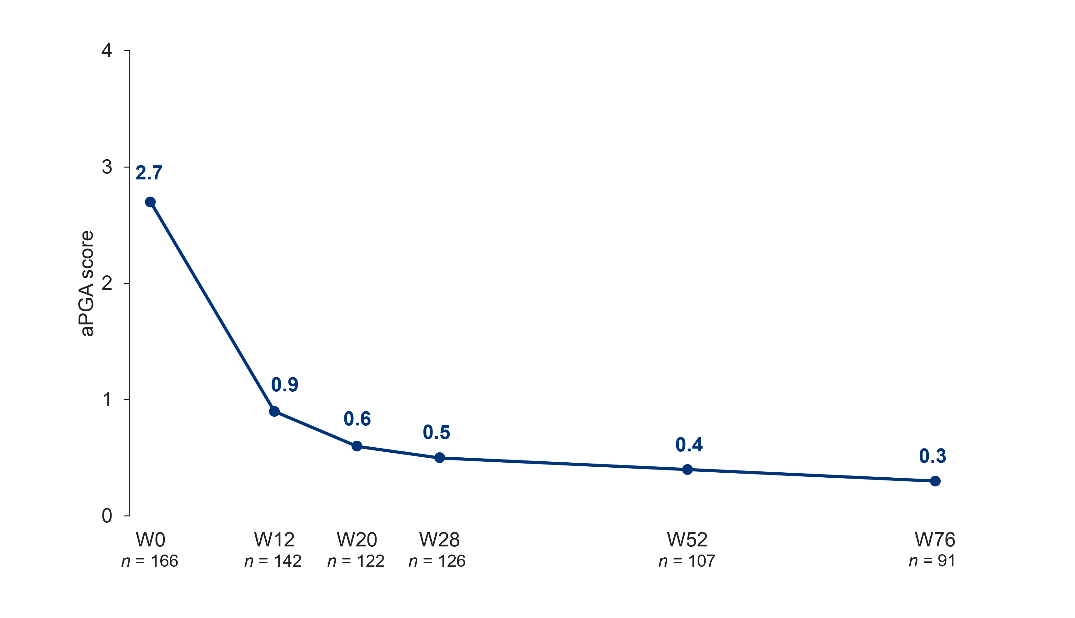


Analysis among patients with aPGA≥1 at baseline.

aPGA, anogenital Physician’s Global Assessment; W, week.

**Figure S6** Mean aPGA score among super-responders, from baseline to week 76


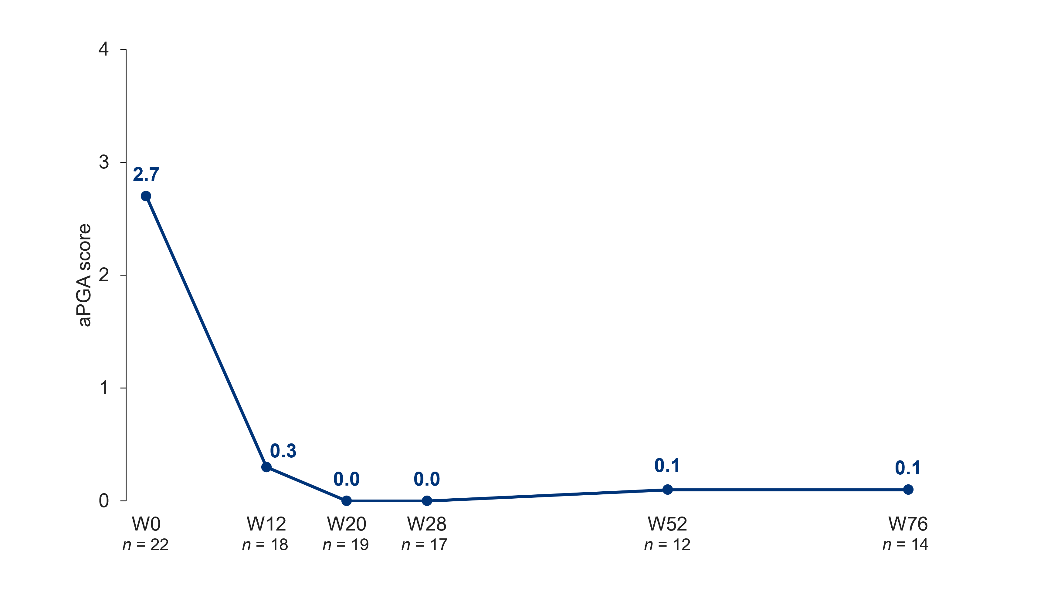


Analysis among patients with aPGA≥1 at baseline.

aPGA, anogenital Physician’s Global Assessment; W, week.

**Figure S7** Mean aPGA scores among BMI subgroups, from baseline to week 76


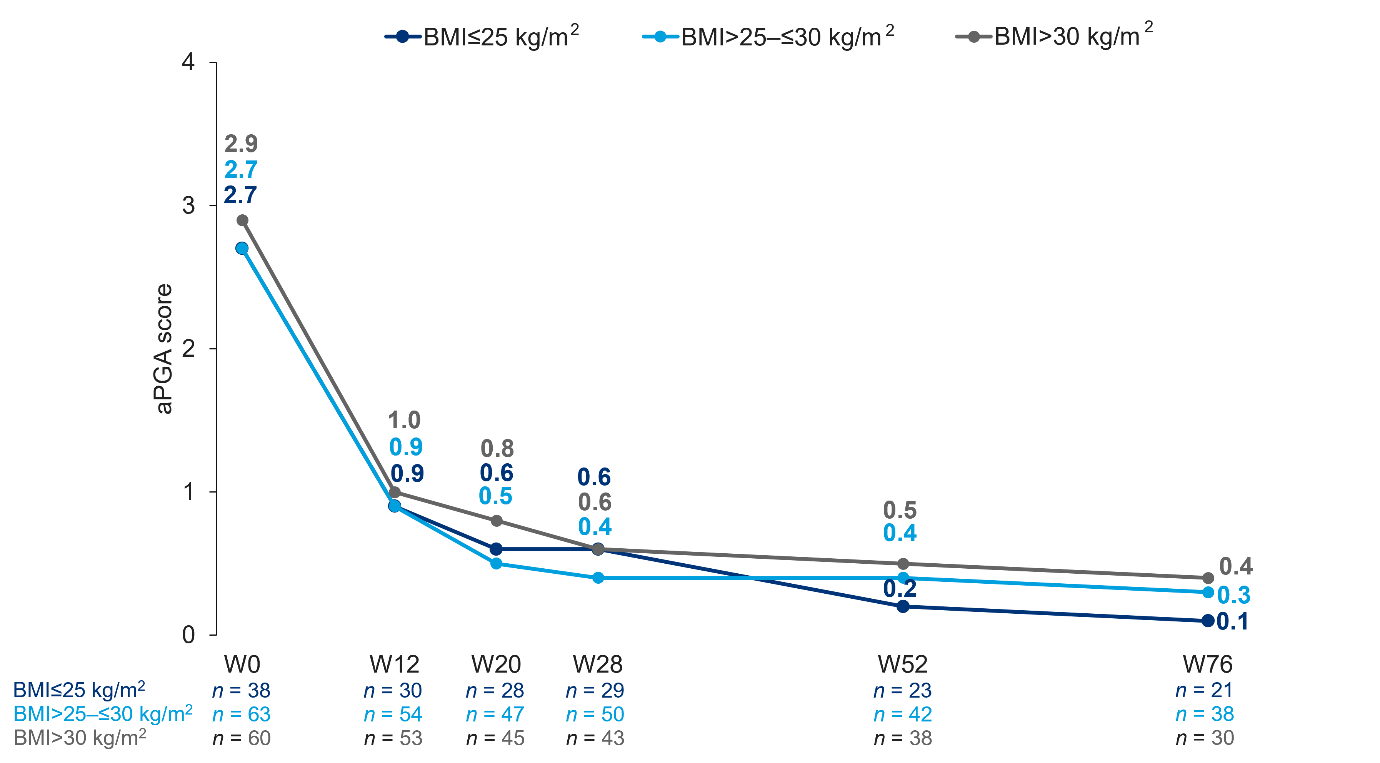


Analysis among patients with aPGA≥1 at baseline.

aPGA, anogenital Physician’s Global Assessment; BMI, body mass index; W, week.

## Dermatology Life Quality Index outcomes

**Figure S8** Mean DLQI from baseline to week 76


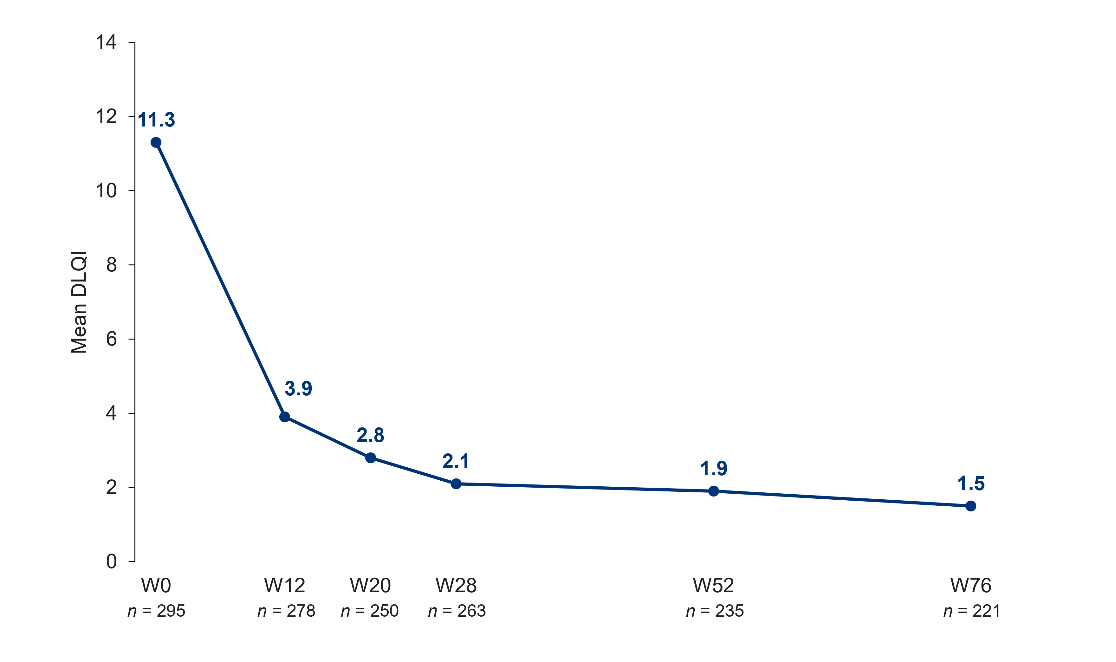


DLQI, Dermatology Life Quality Index; W, week.

**Figure S9** Mean DLQI from baseline to week 76 among patients who had aPGA≤1 or aPGA>1 at week 28


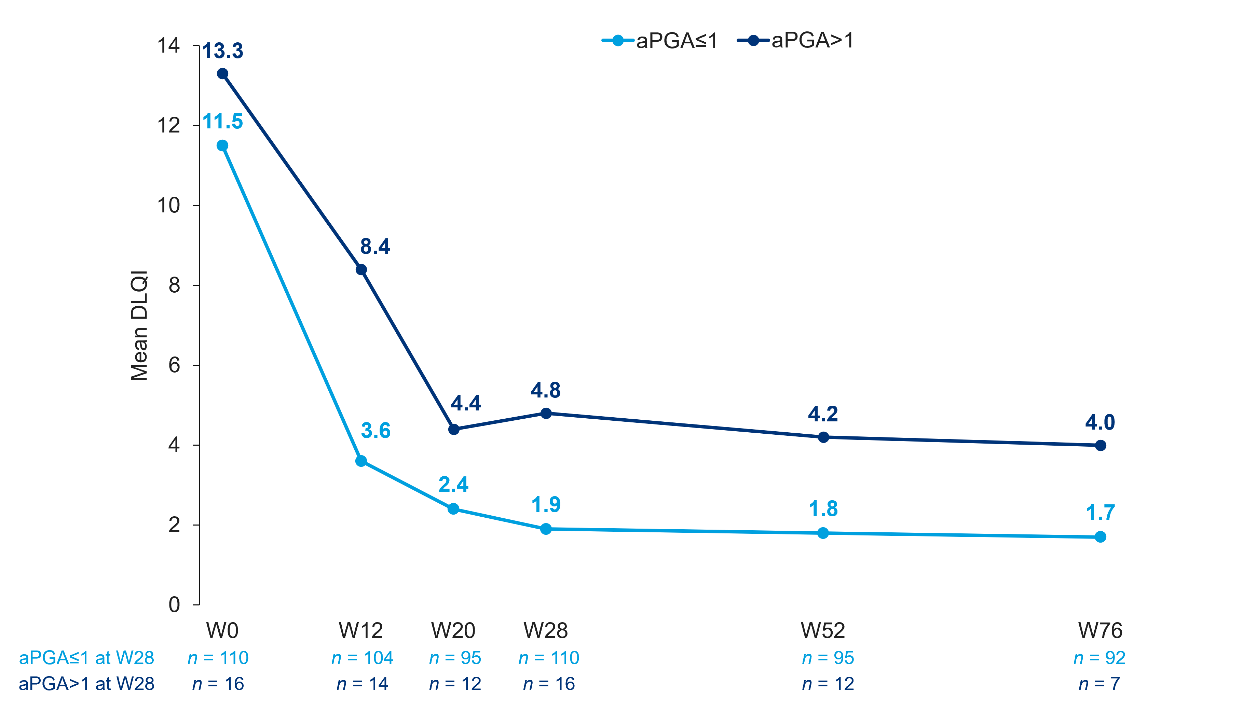


aPGA, anogenital Physician’s Global Assessment; DLQI, Dermatology Life Quality Index; W, week.

**Figure S10** Mean DLQI among subgroups of patients defined by disease duration, from baseline to week 76


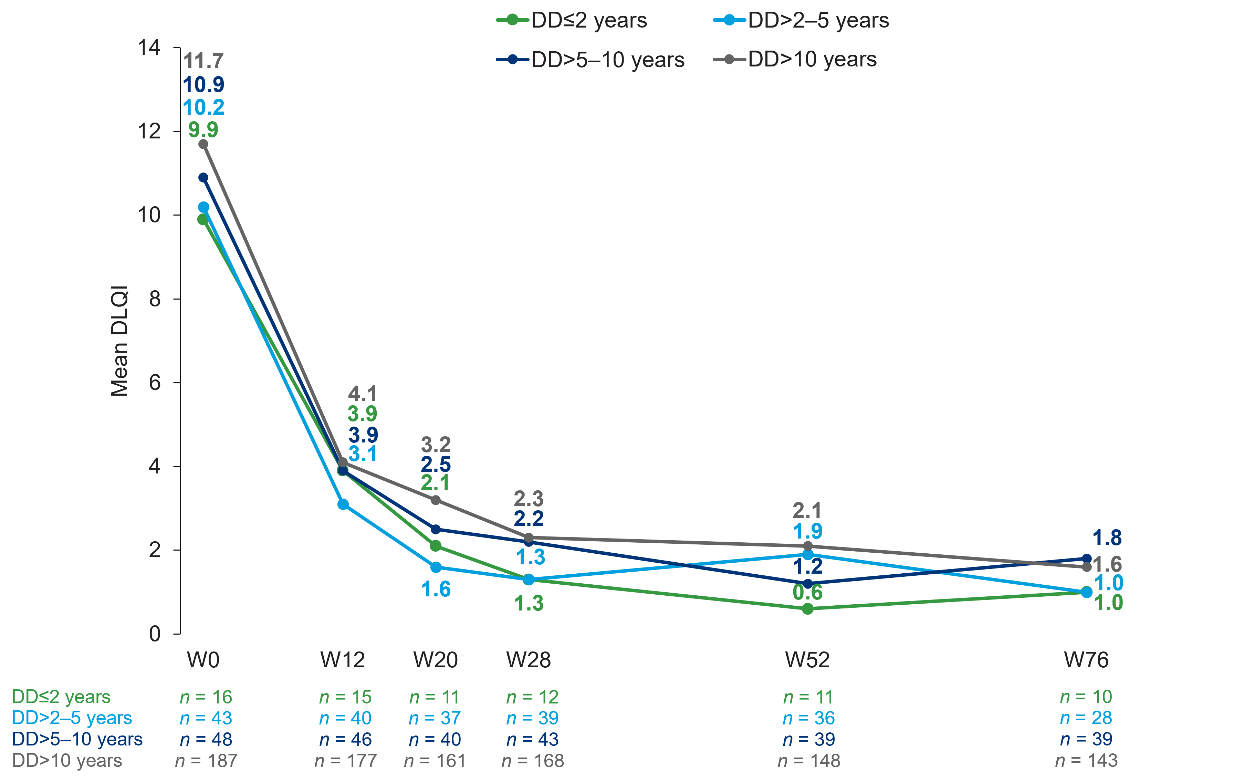


DD, disease duration; DLQI, Dermatology Life Quality Index; W, week.

**Figure S11** Individual DLQI question responses from baseline to week 76


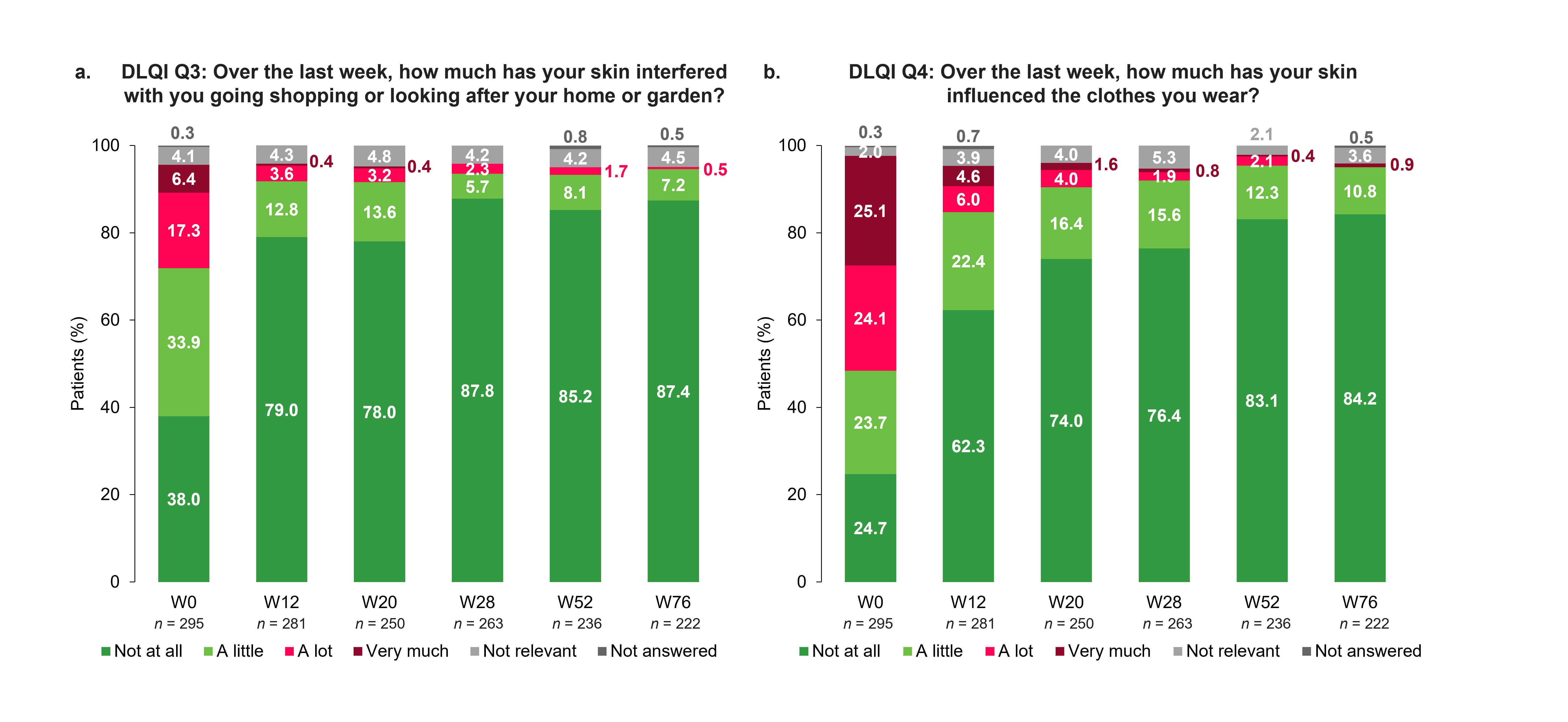


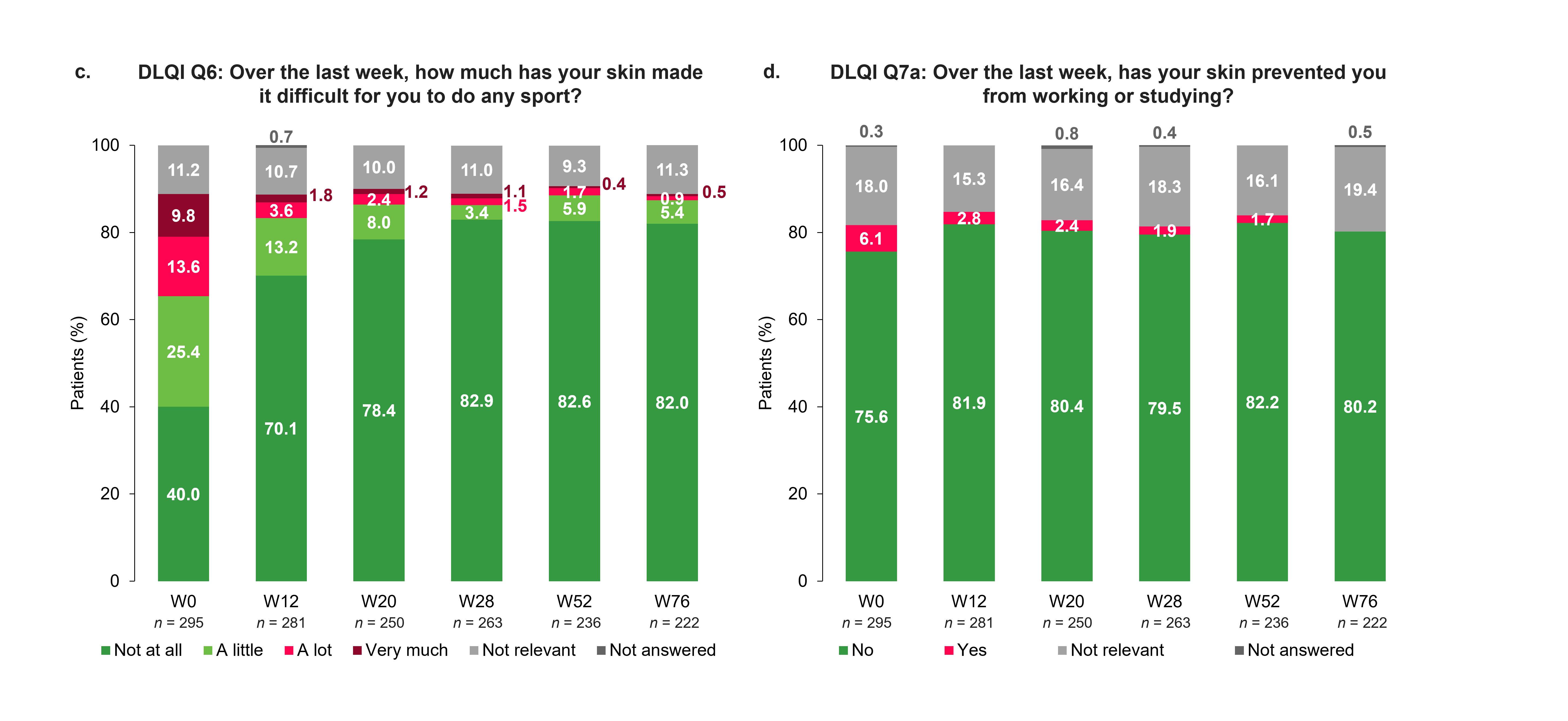


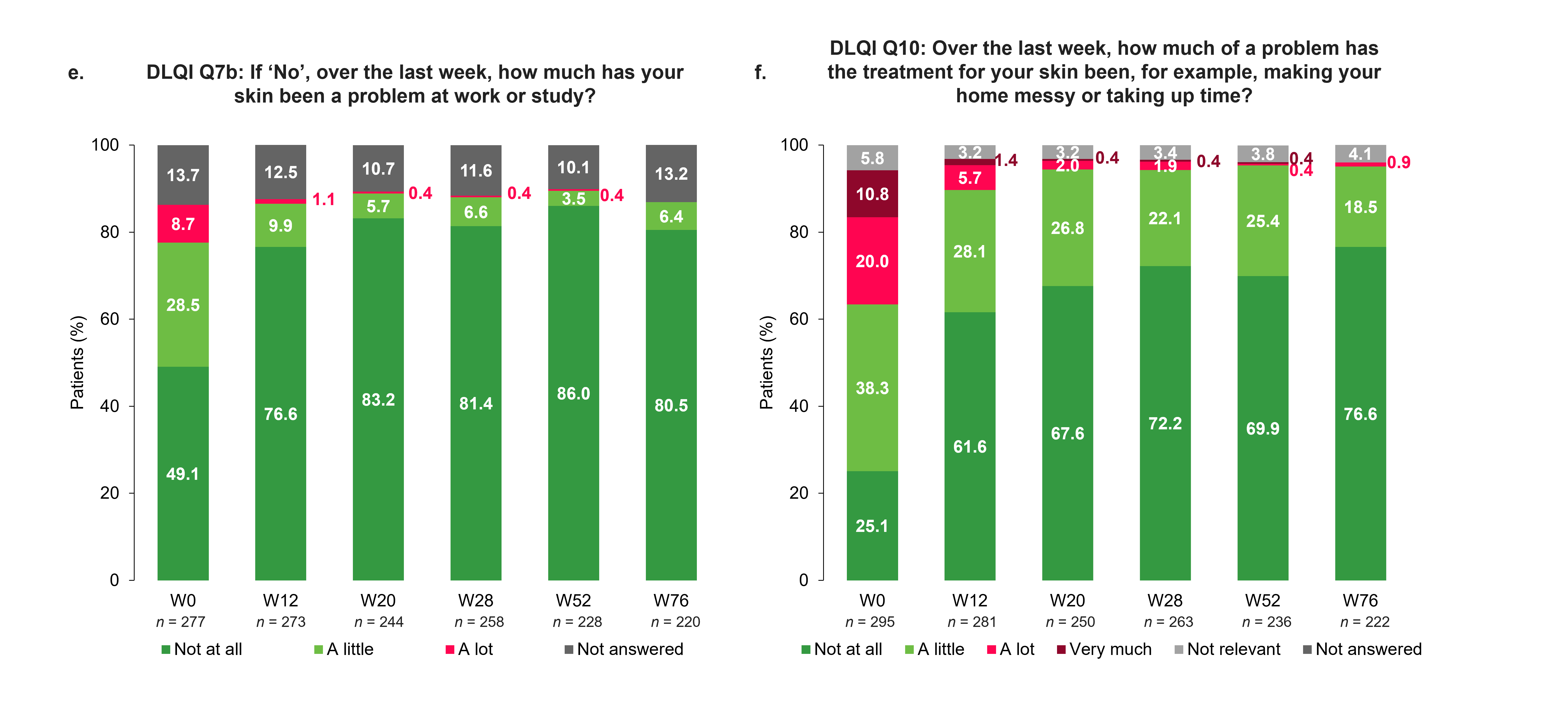


Individual DLQI questions showing patient responses from baseline to W76 for (a) Q3, (b) Q4, (c) Q6, (d) Q7a, (e) Q7b, and (f) Q10. If response to Q7a was ‘no’ or ‘not relevant’, or the question was not answered, responses to Q7b were analyzed.

DLQI, Dermatology Life Quality Index; Q, Question; W, week.

## Relationship and Sexuality Scale outcomes

**Figure S12** Individual RSS question responses from baseline to week 76


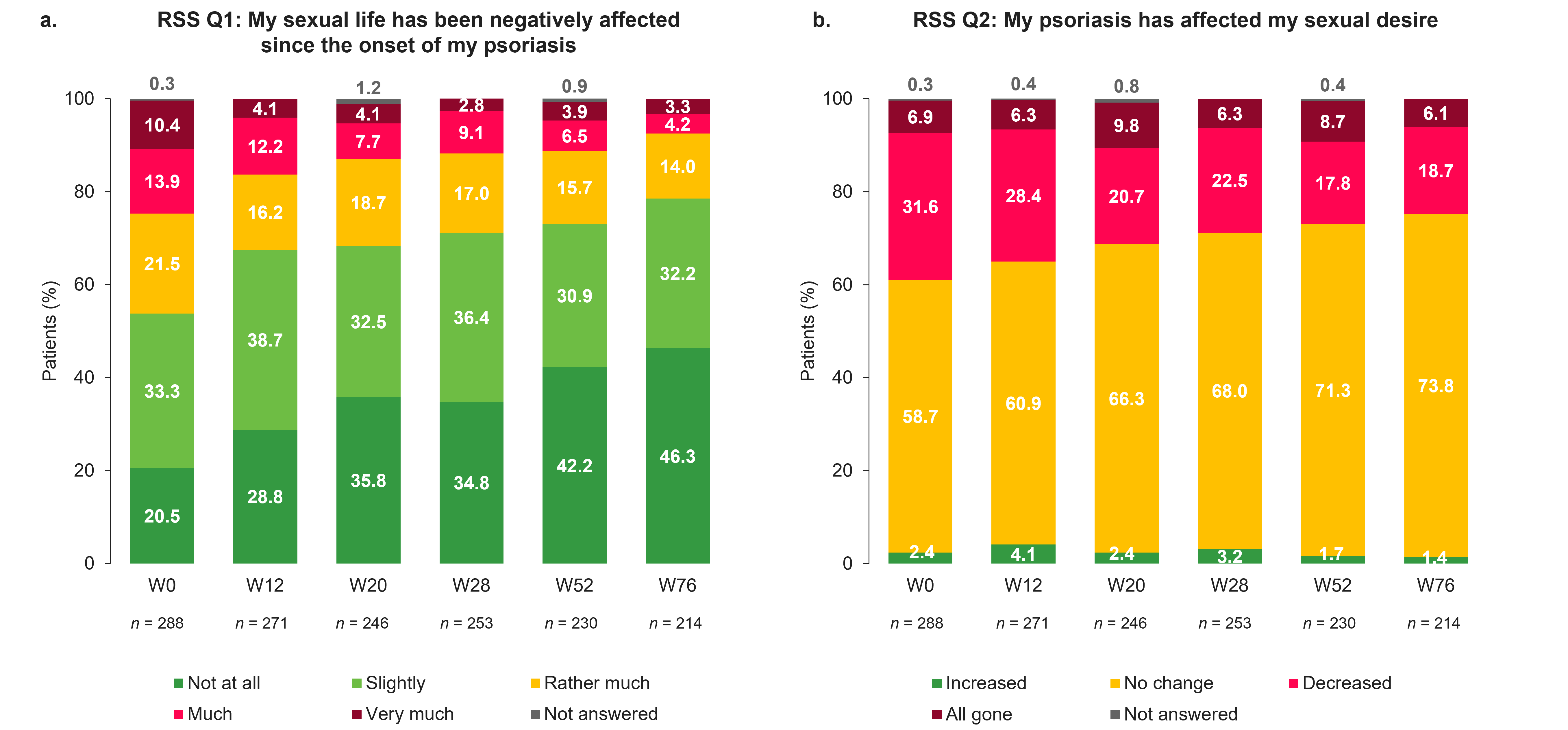


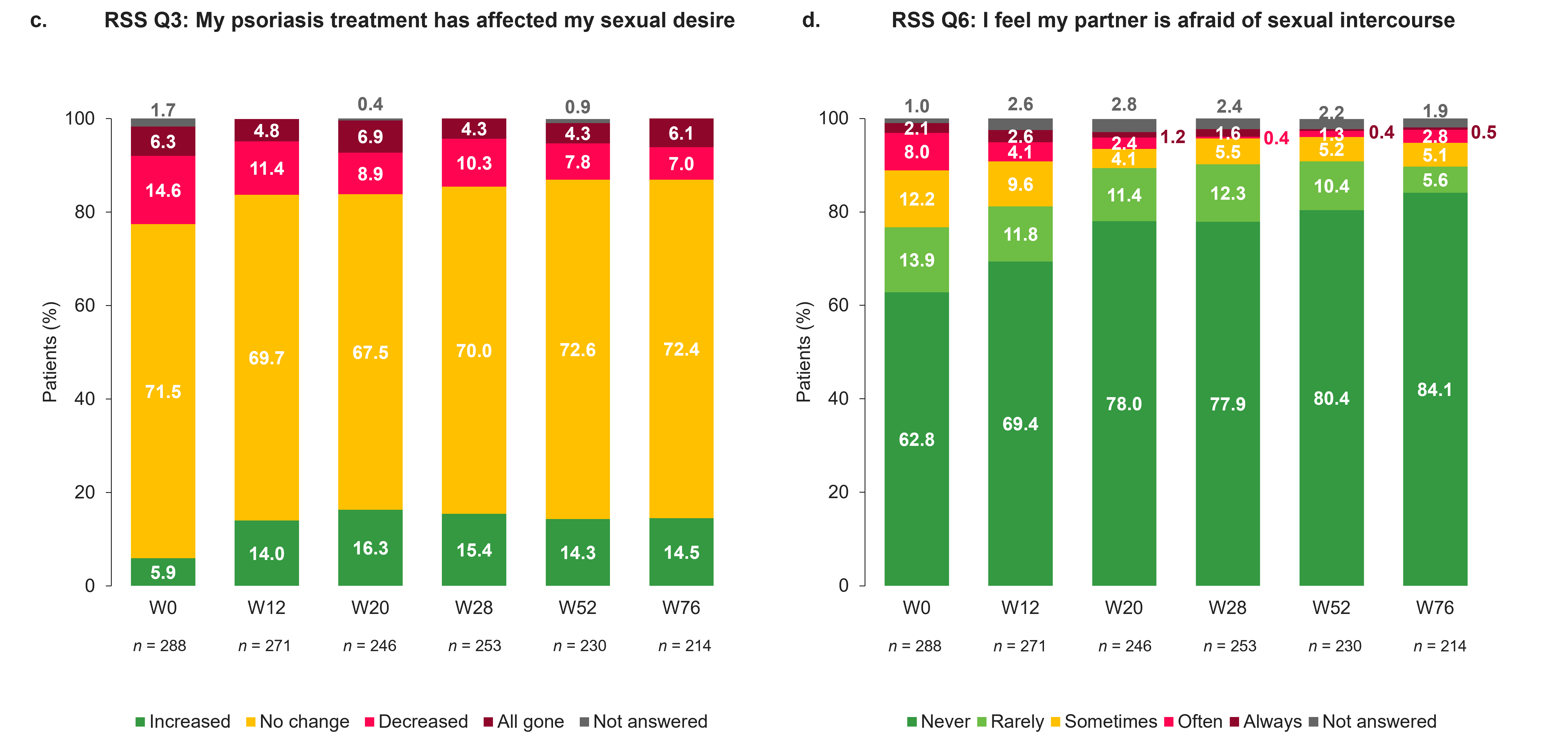


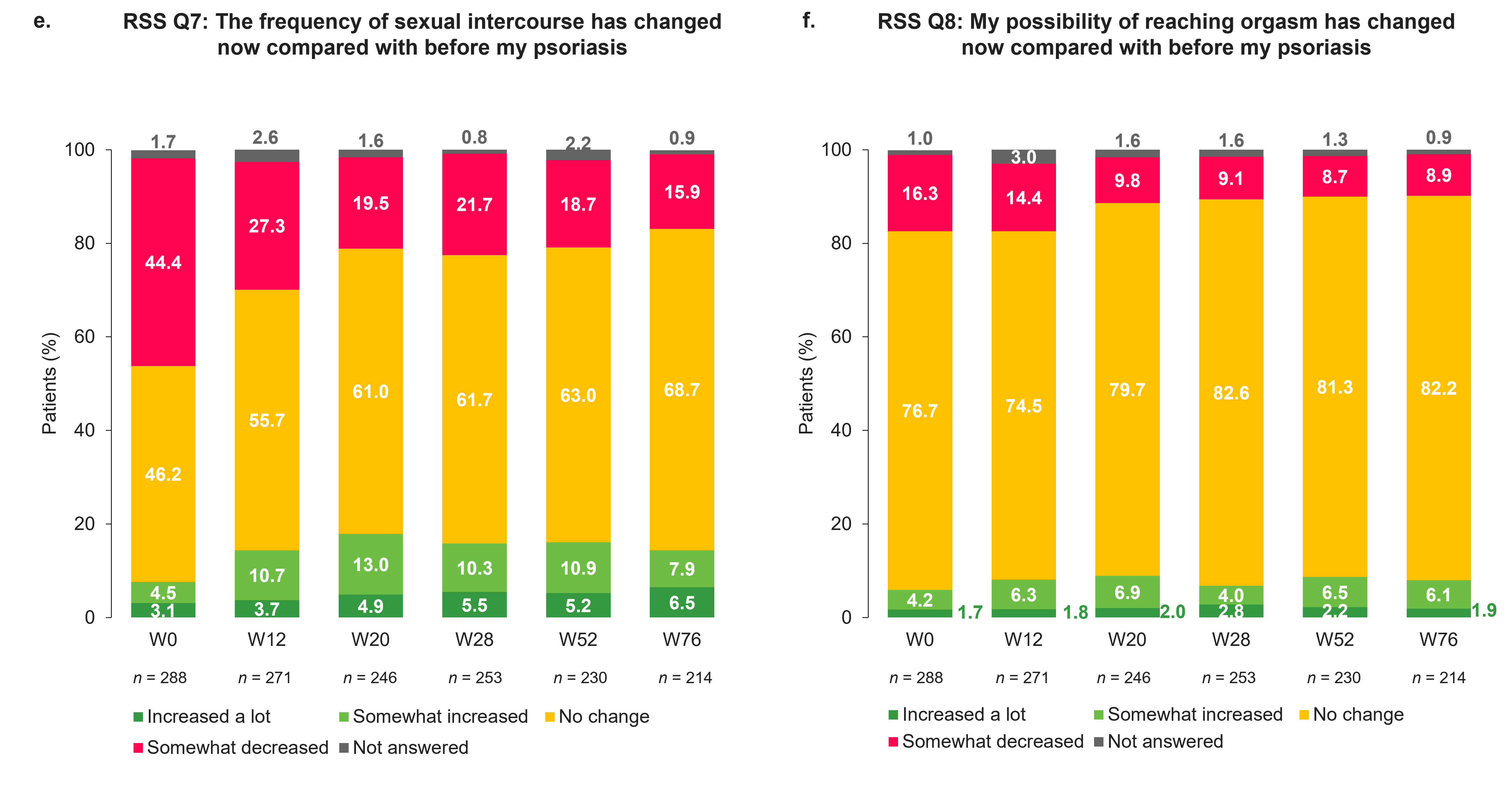


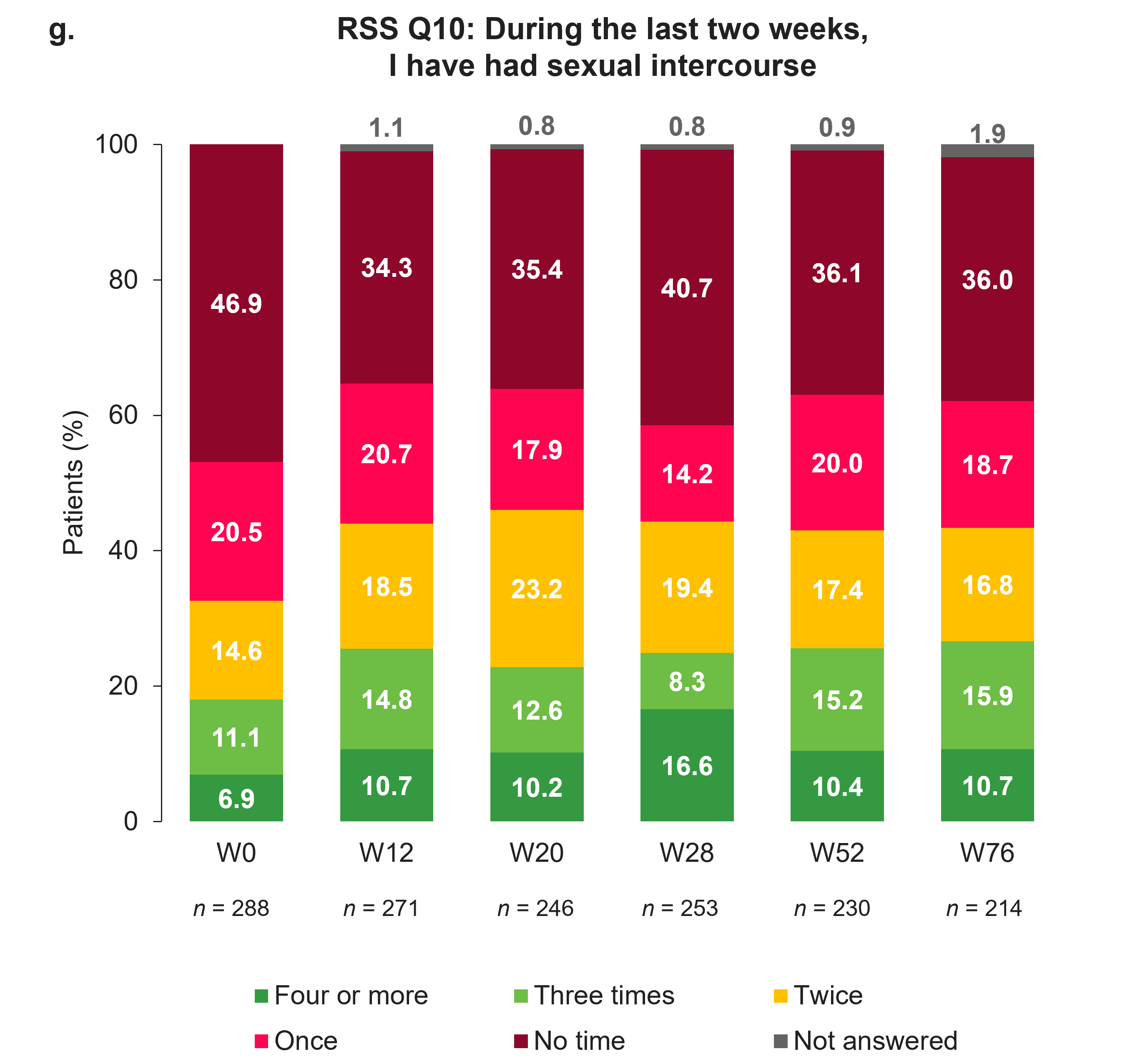


Individual RSS questions showing patient responses from baseline to W76 for (a) Q1, (b) Q2, (c) Q3, (d) Q6, (e) Q7, (f) Q8, and (g) Q10.

Q, Question; RSS, Relationship and Sexuality Scale; W, week.

**Figure S13** RSS Q4 responses from baseline to week 76 by different patient subgroups


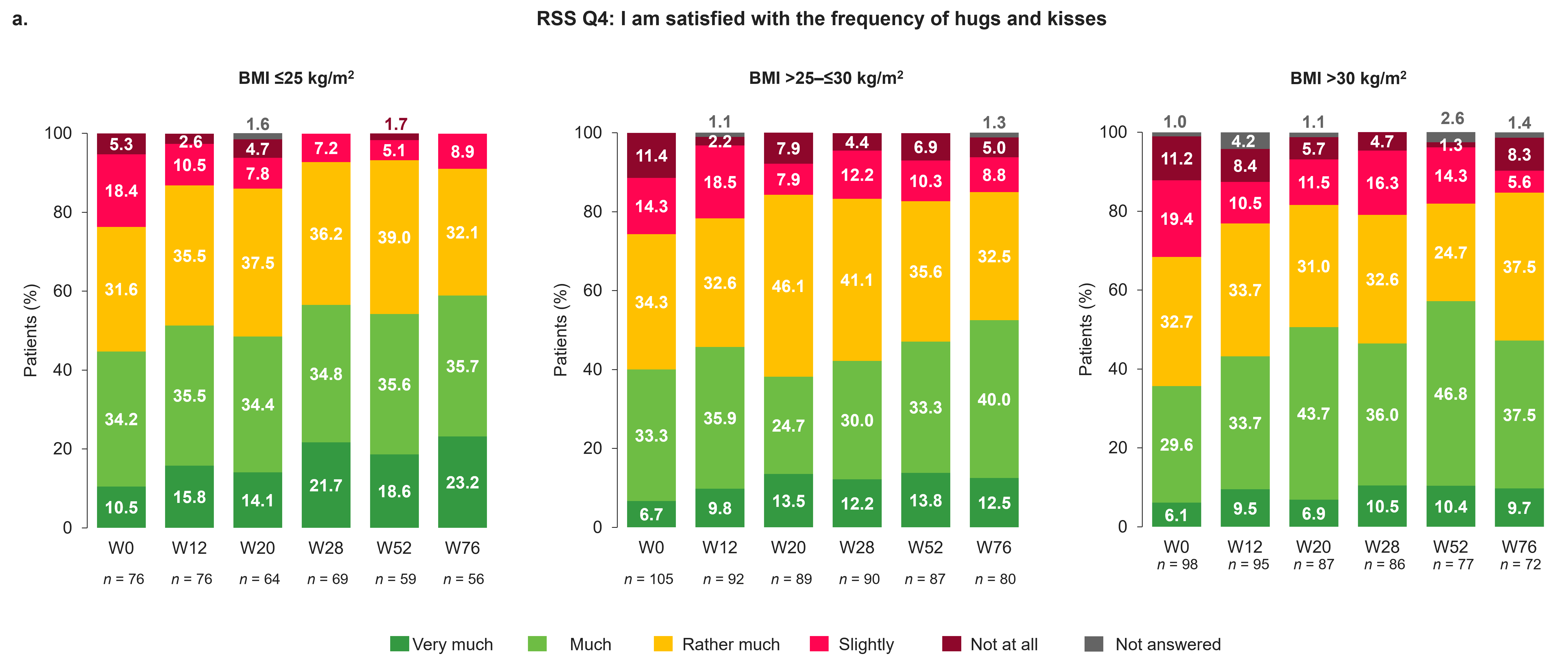


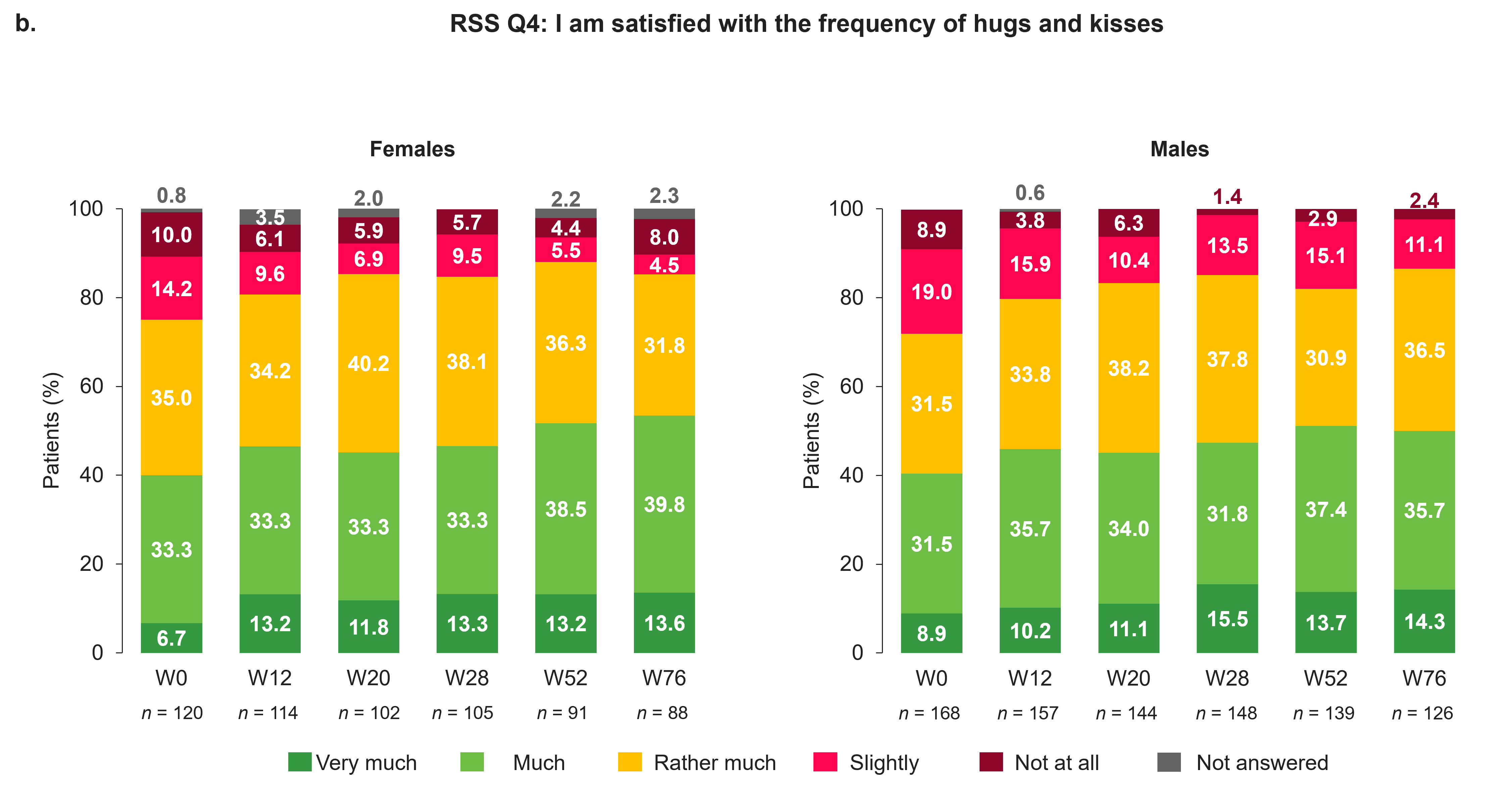


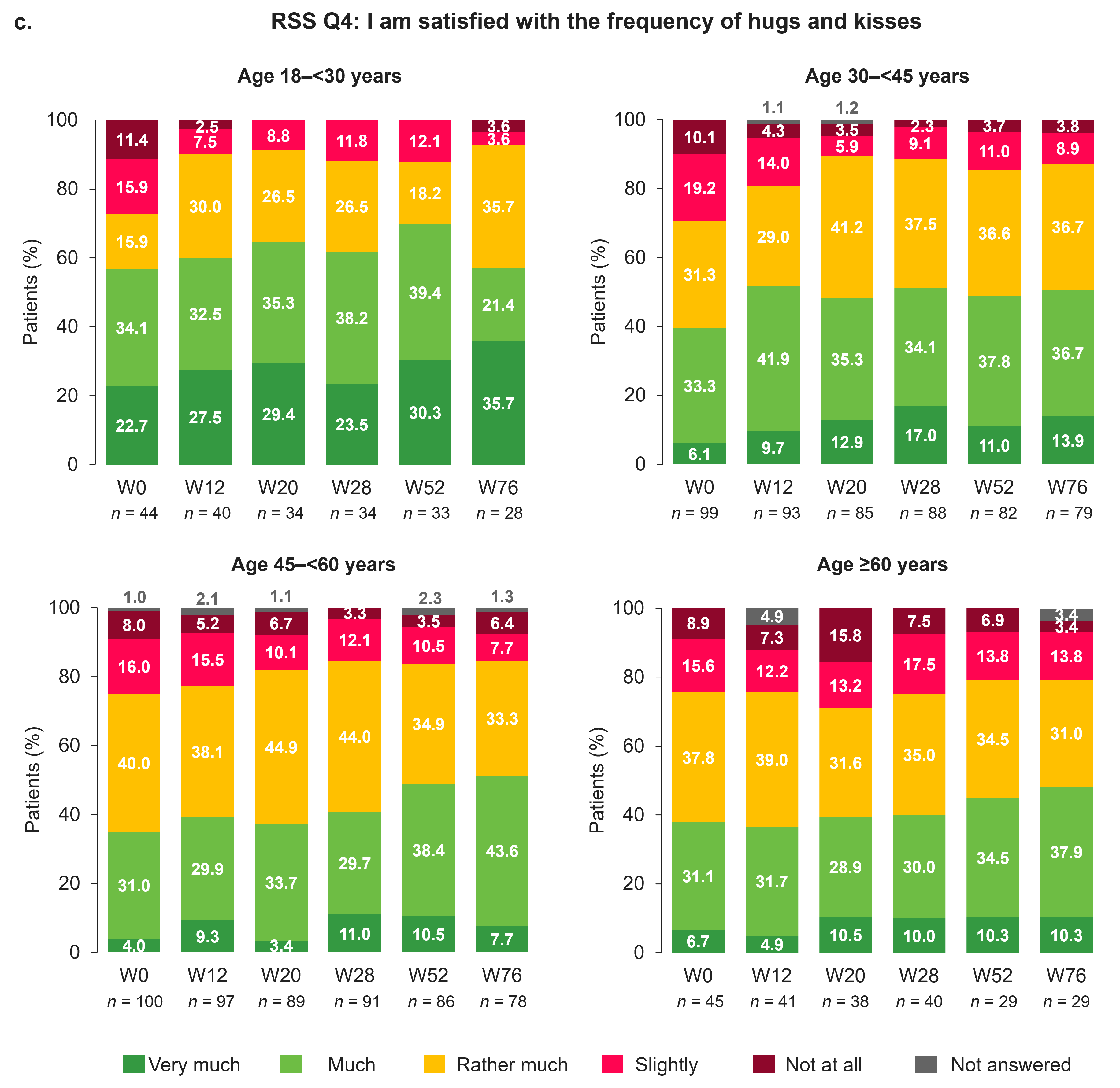


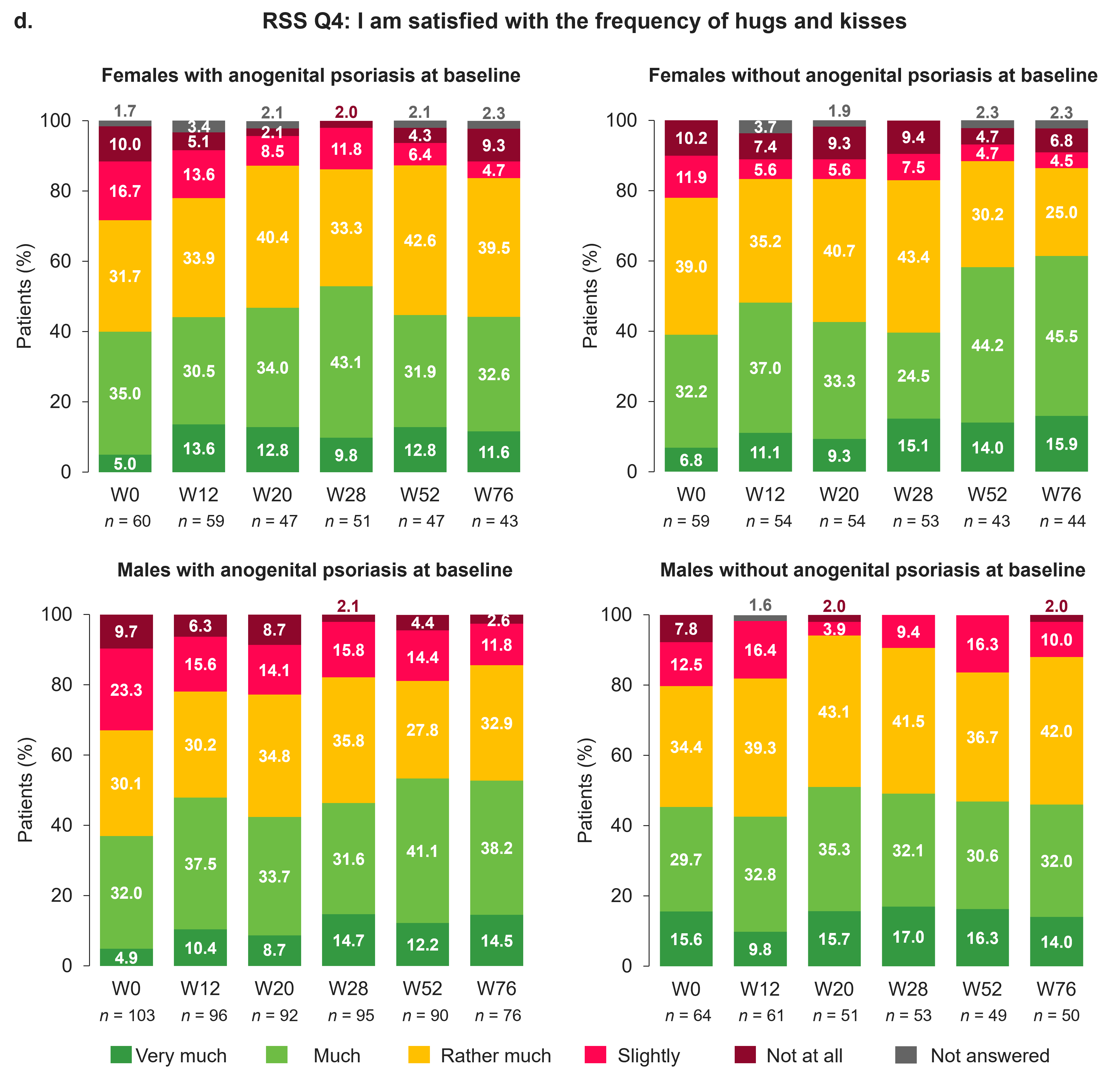


RSS Q4 responses from baseline to W76 in subgroups of patients defined by (a) BMI (b) sex, (c) age, and (d) sex with (aPGA≥1) and without anogenital psoriasis at baseline.

aPGA, anogenital Physician’s Global Assessment; BMI, body mass index; Q, Question; RSS, Relationship and Sexuality Scale; W, week.

**Figure S14** RSS Q5 responses from baseline to week 76 by different patient subgroups


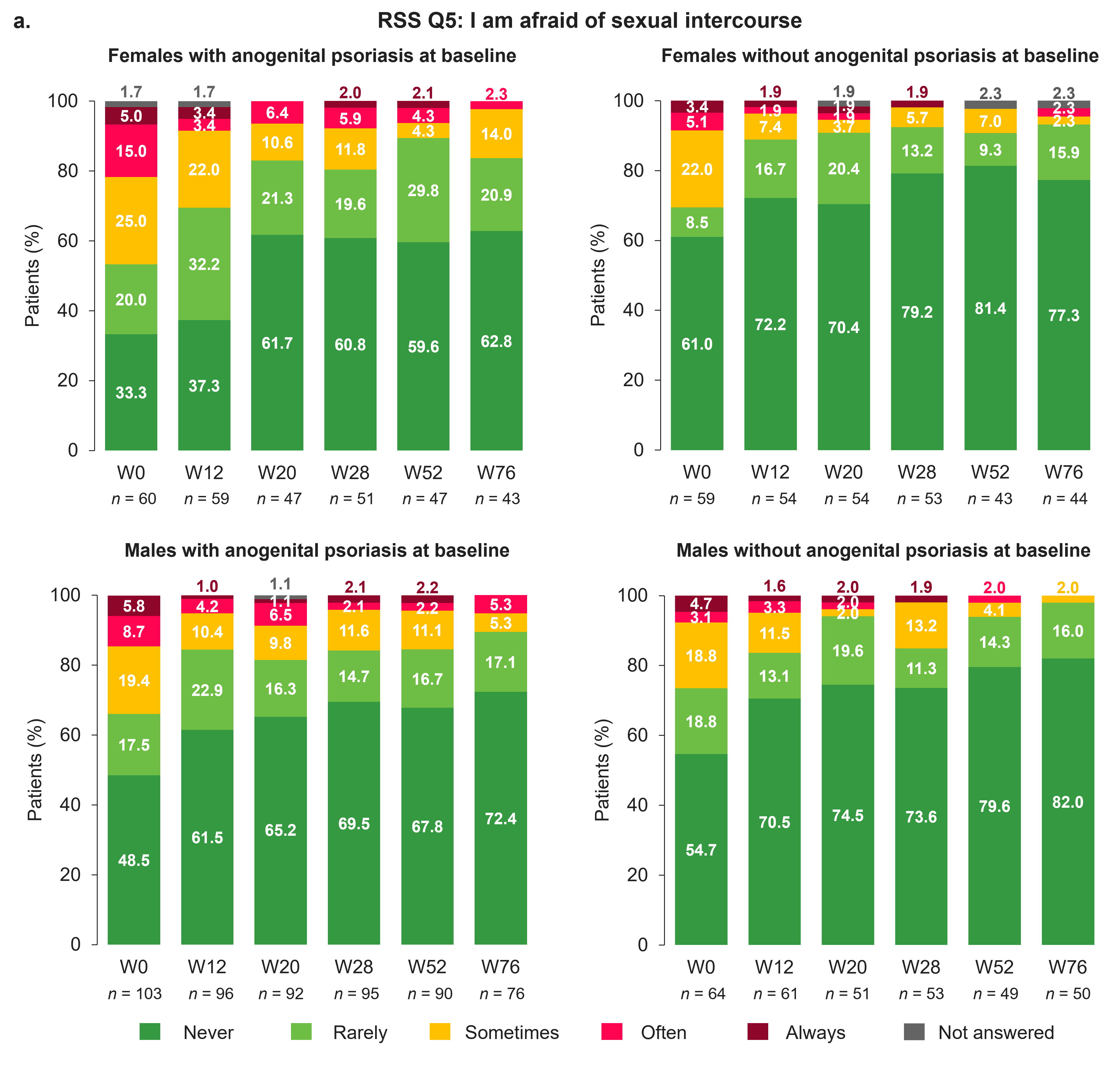


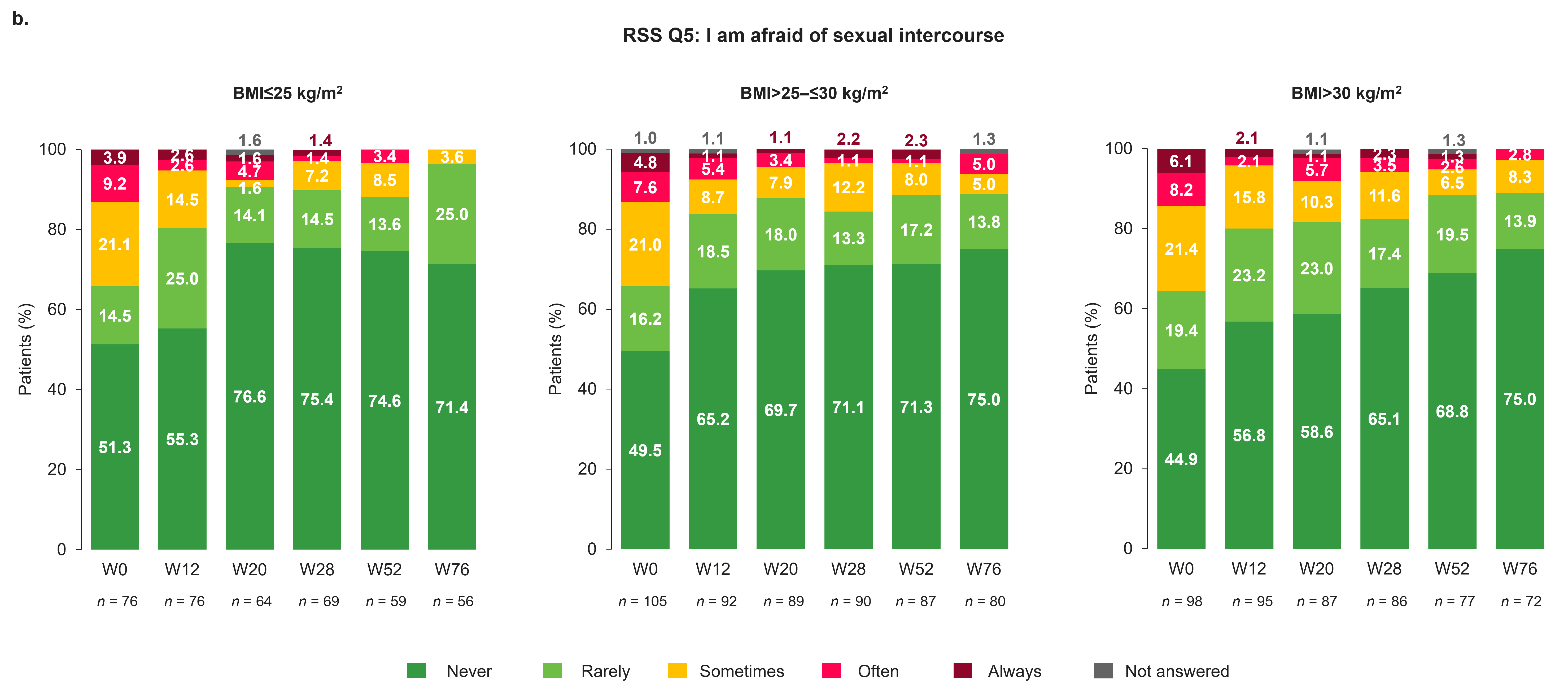


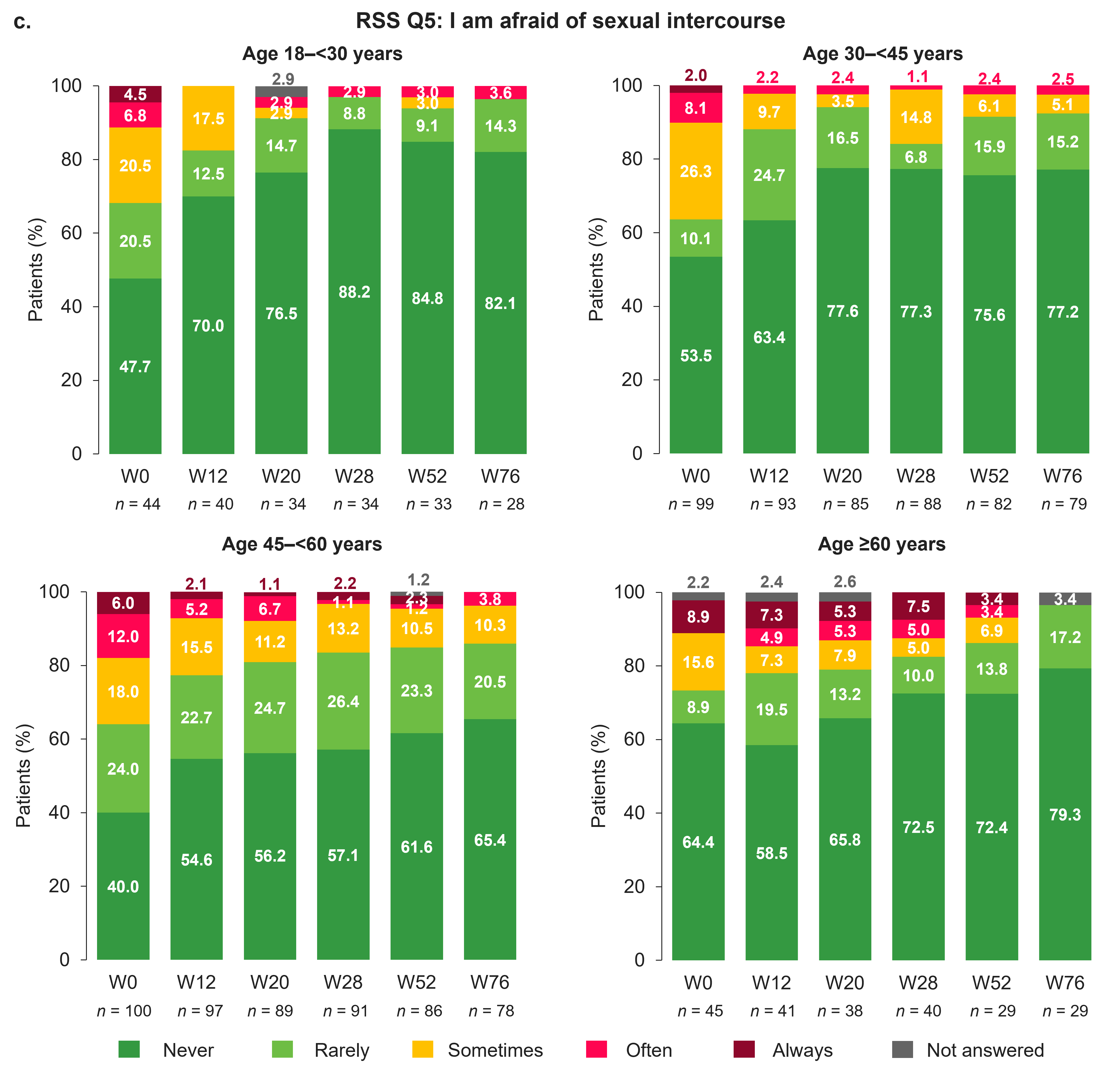


RSS Q5 responses from baseline to W76 in subgroups of patients defined by (a) sex with (aPGA≥1) and without anogenital psoriasis at baseline, (b) BMI, and (c) age.

aPGA, anogenital Physician’s Global Assessment; BMI, body mass index; Q, Question;
RSS, Relationship and Sexuality Scale; W, week.

**Figure S15** RSS Q9 responses from baseline to week 76 by different patient subgroups


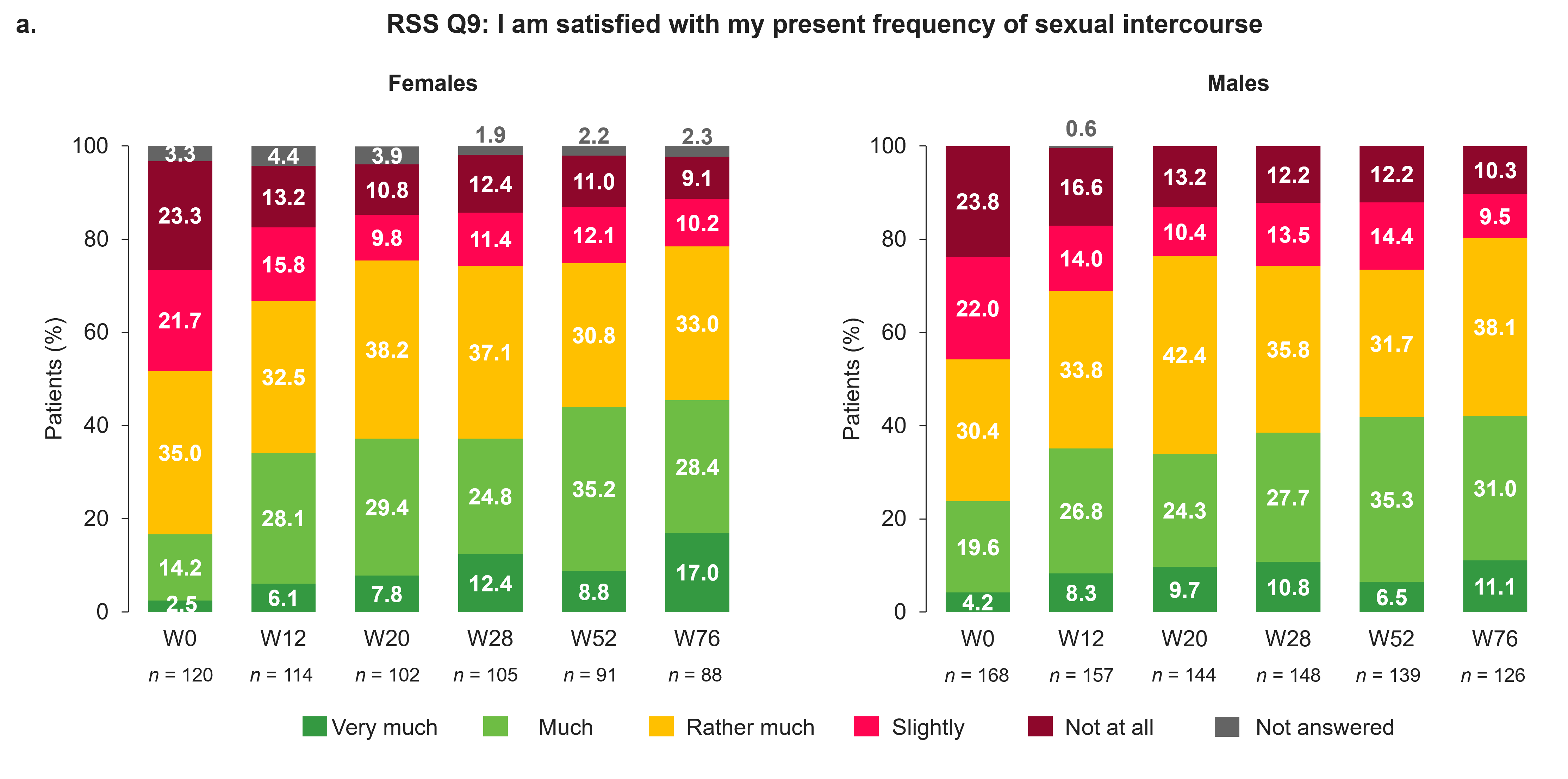


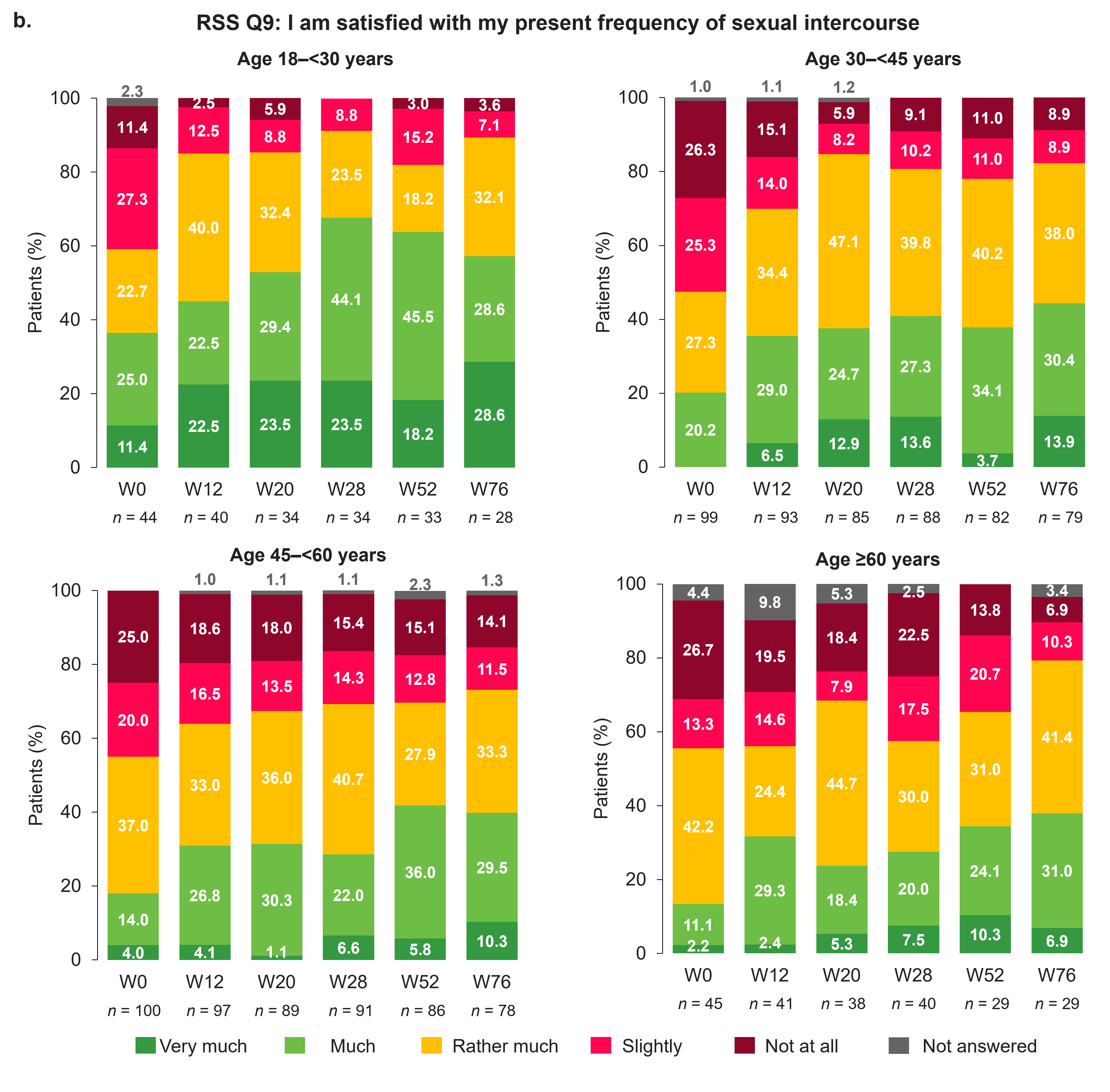


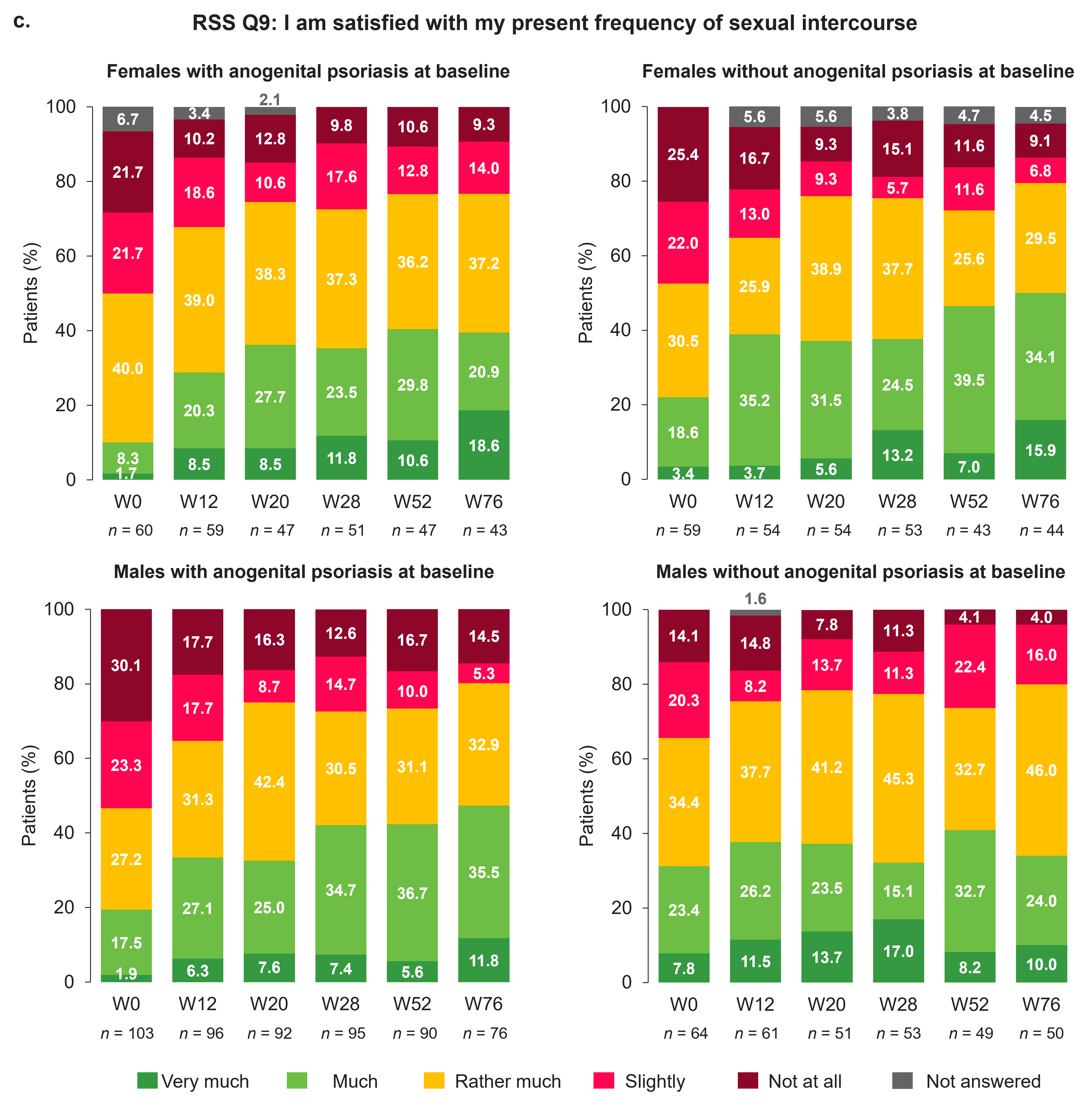


RSS Q9 responses from baseline to W76 in subgroups of patients defined by (a) sex, (b) age, and
(c) sex with (aPGA≥1) and without anogenital psoriasis at baseline.

aPGA, anogenital Physician’s Global Assessment; Q, Question; RSS, Relationship and Sexuality Scale; W, week.

## Perceived Stigmatization Questionnaire outcomes

**Figure S16** Individual PSQ question responses from baseline to week 76


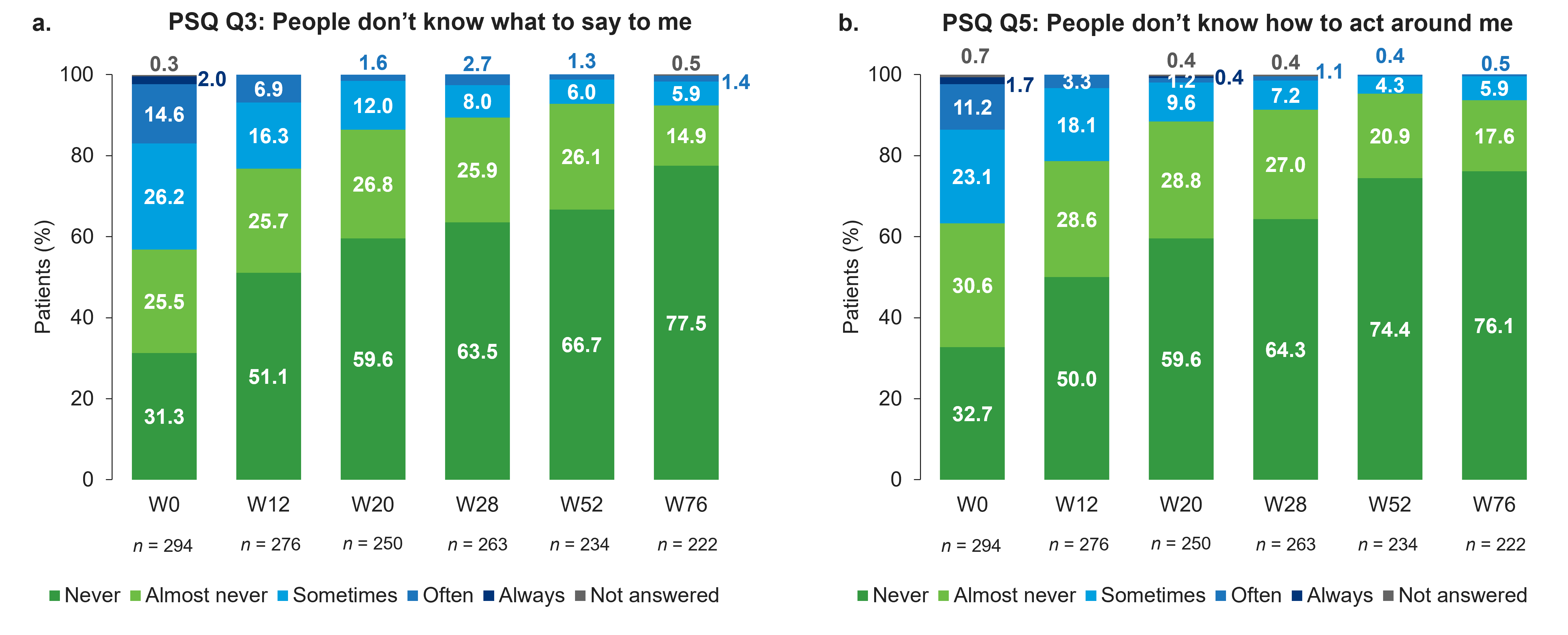


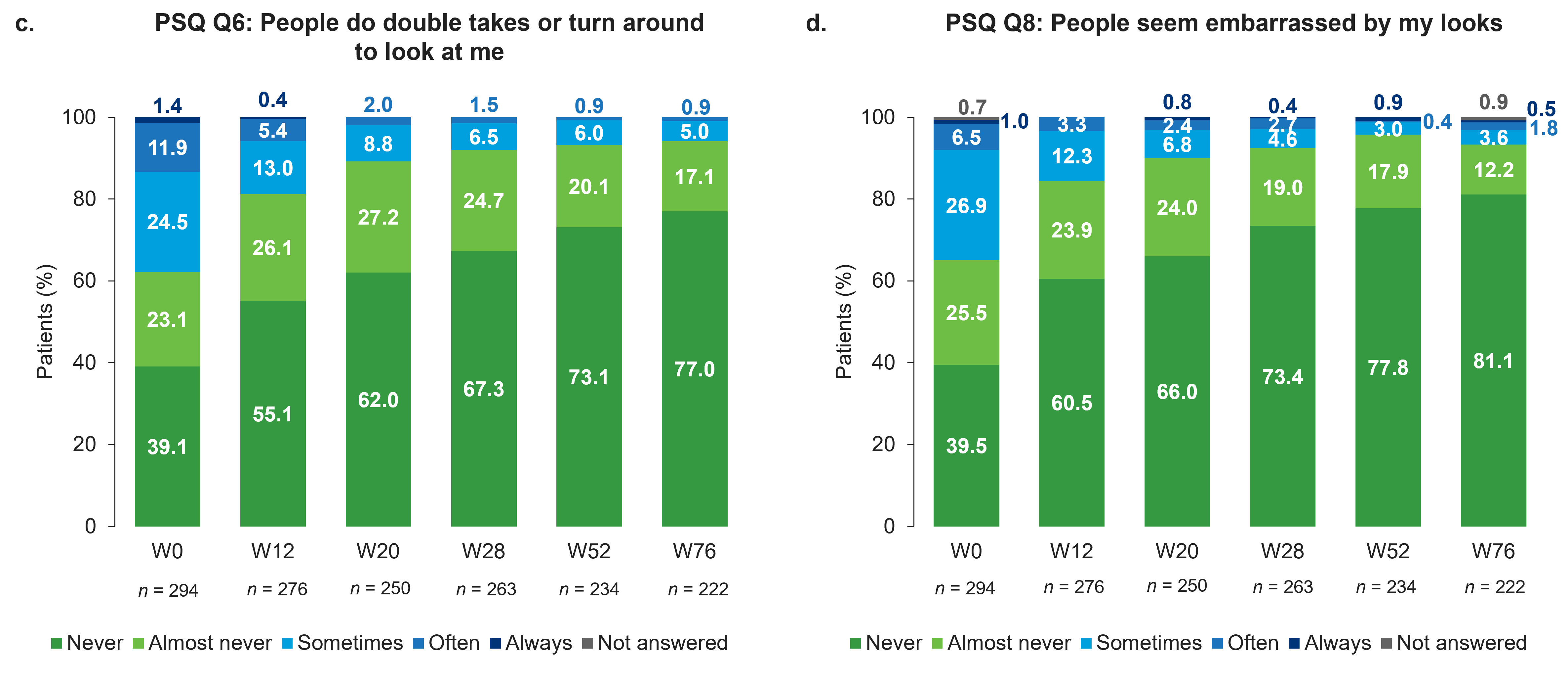


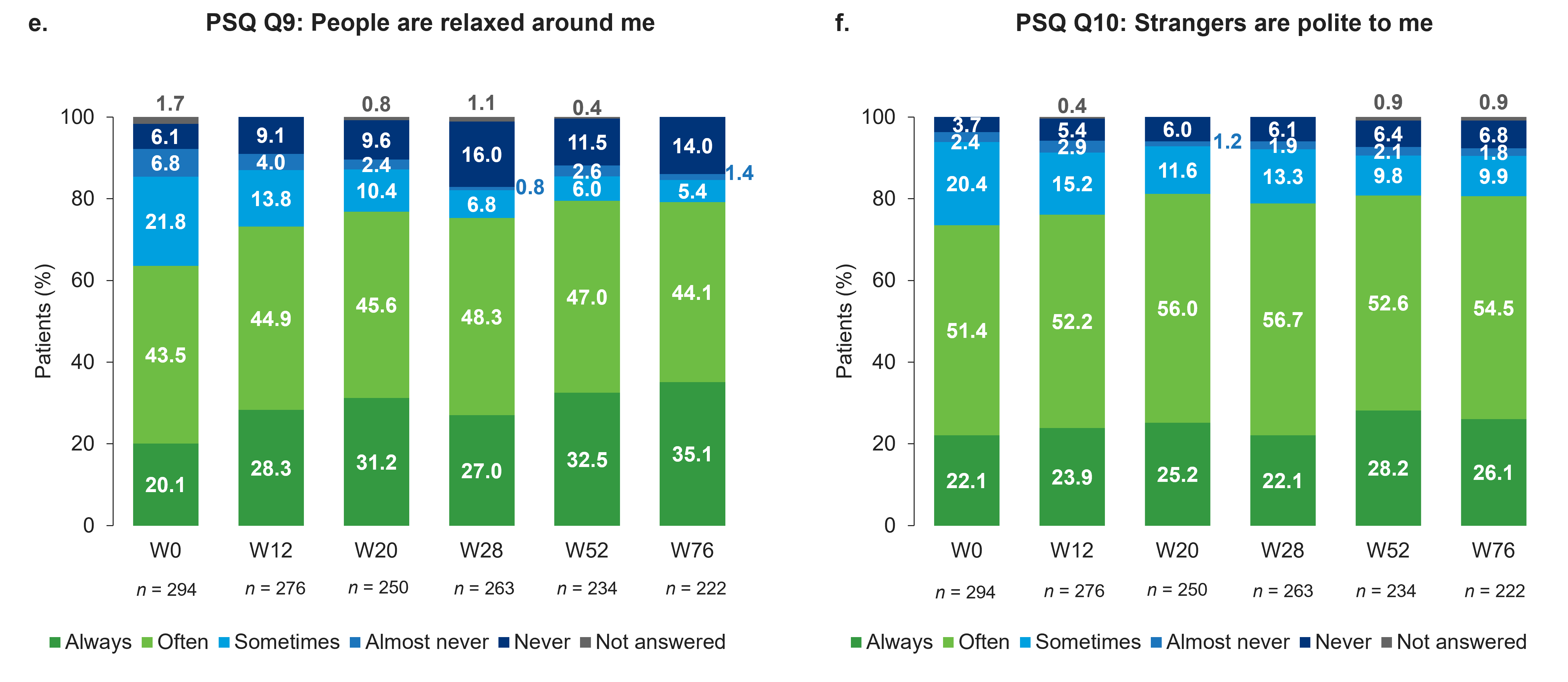


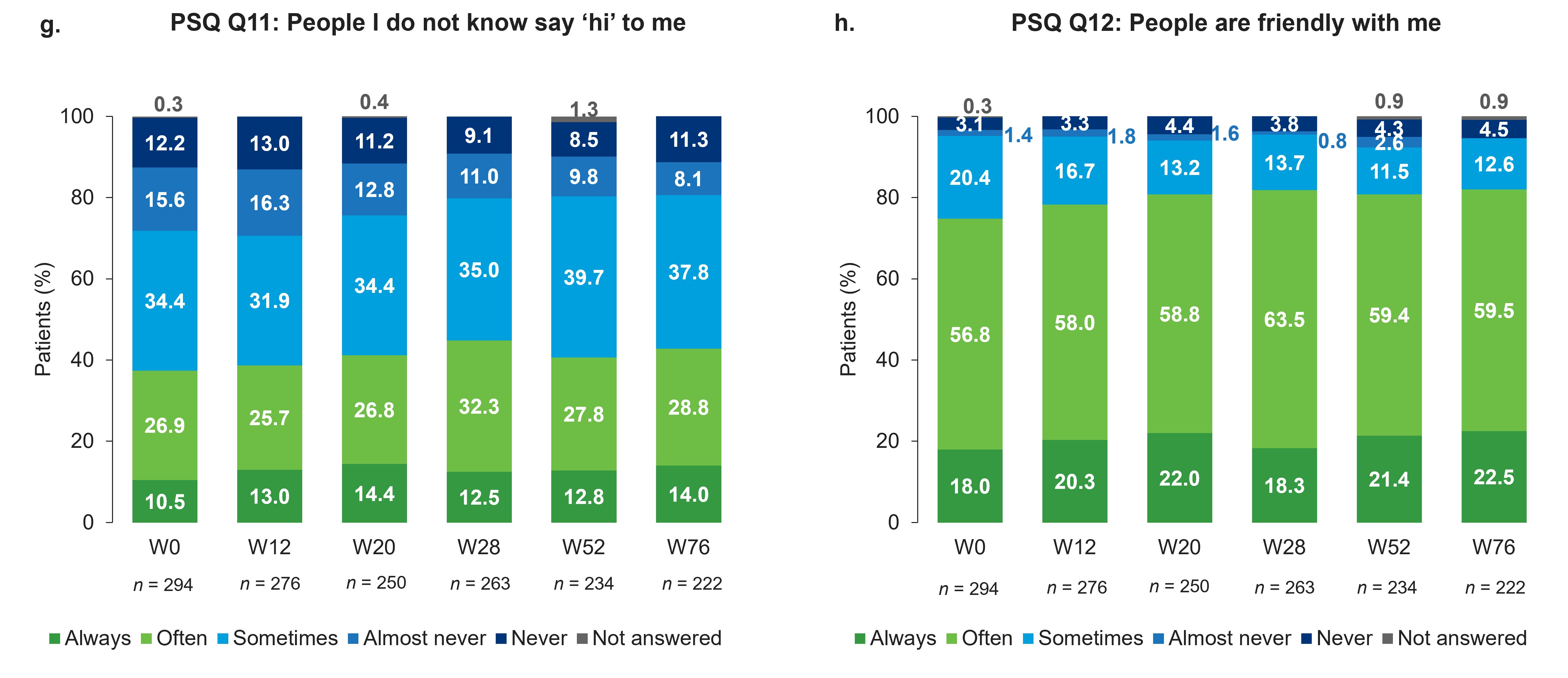


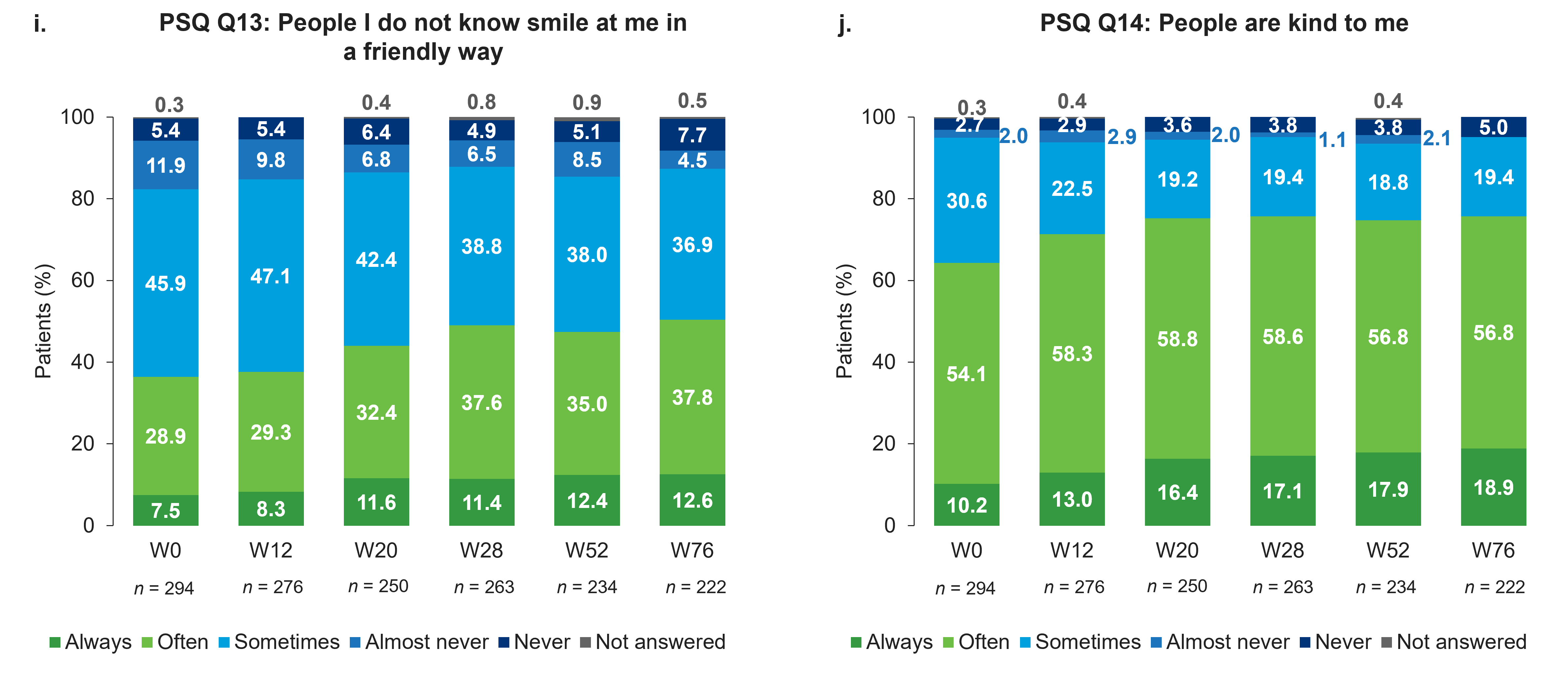


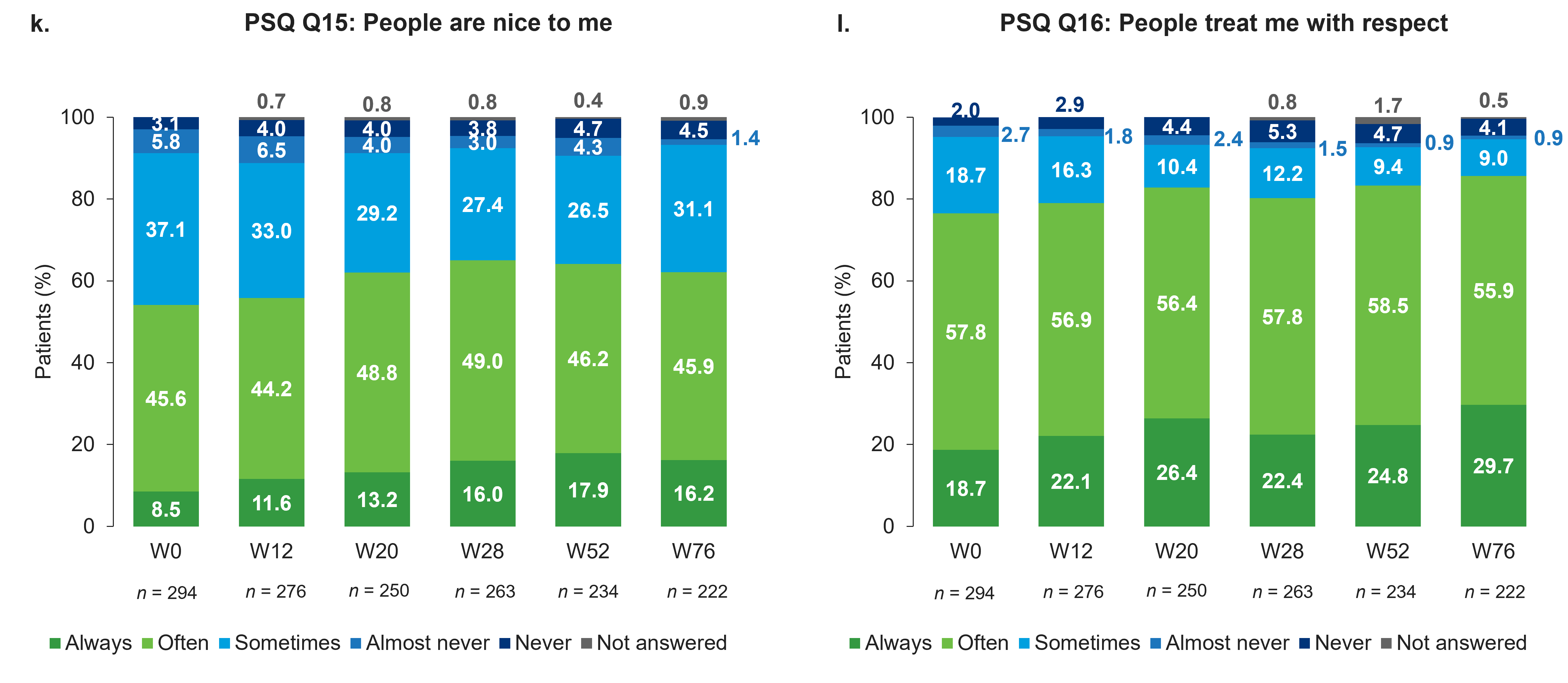


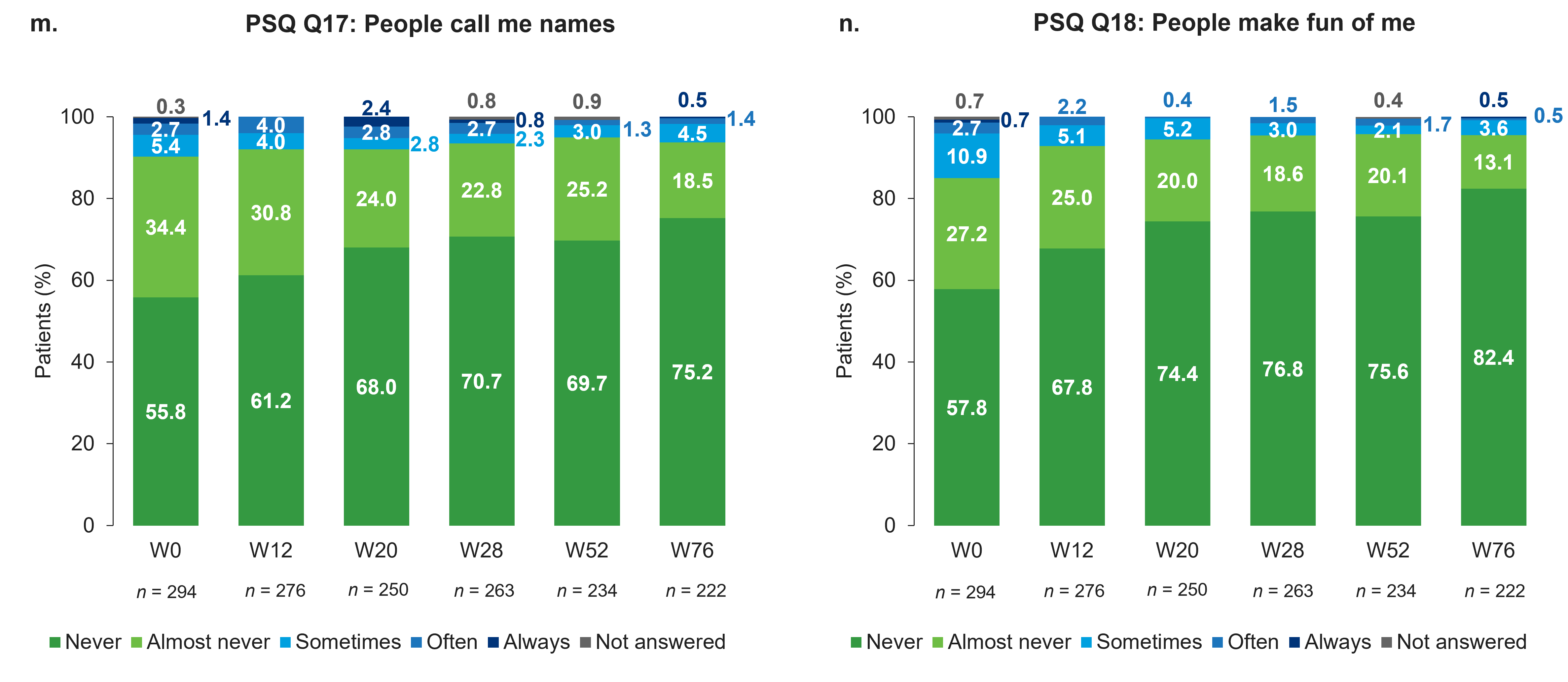


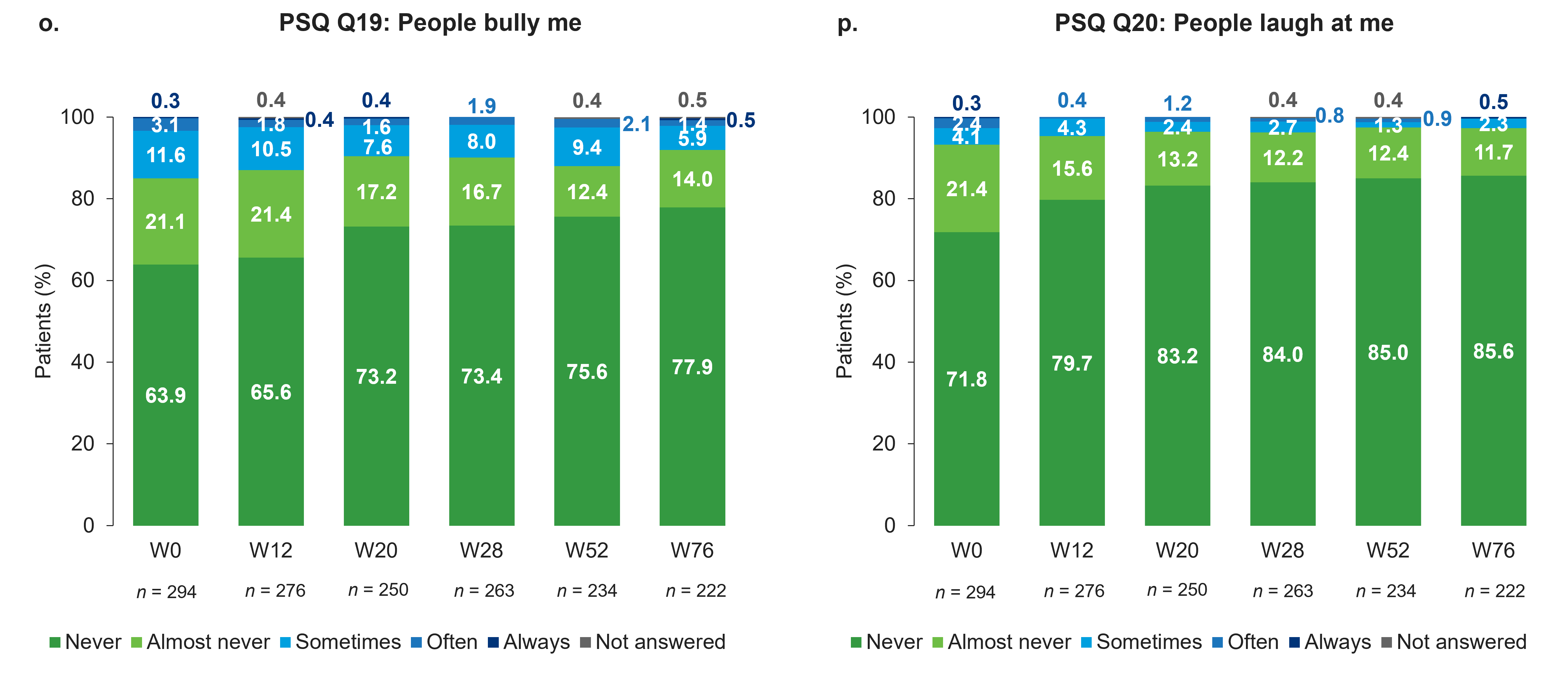


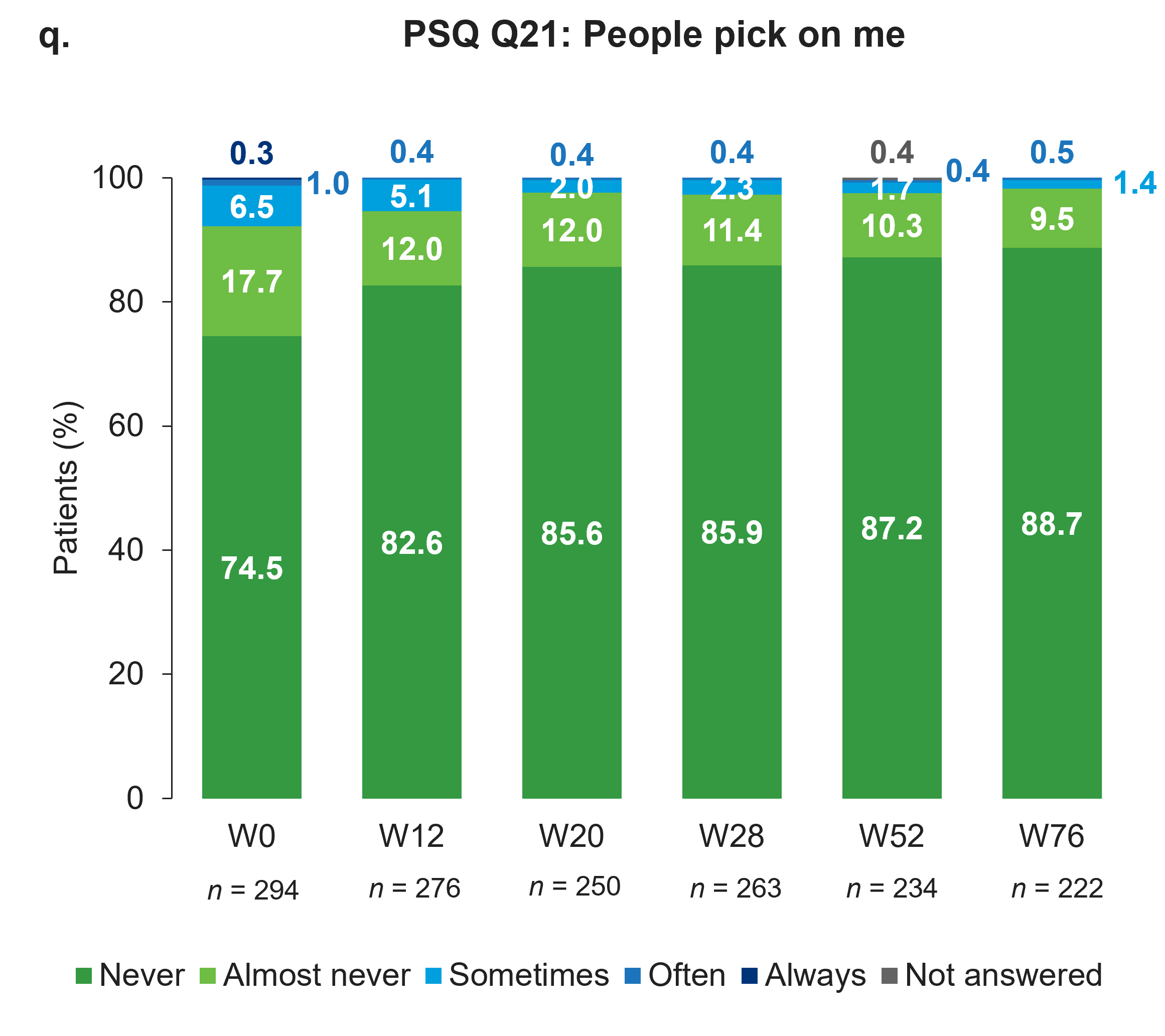


Individual PSQ questions showing patient responses from baseline to W76 for (a) Q3, (b) Q5, (c) Q6, (d) Q8, (e) Q9, (f) Q10, (g) Q11, (h) Q12, (i) Q13, (j) Q14, (k) Q15, (l) Q16, (m) Q17, (n) Q18, (o) Q19, (p) Q20, and (q) Q21.

PSQ, Perceived Stigmatization Questionnaire; Q, Question; W, week.

**Figure S17** PSQ Q2, Q4, and Q7 responses from baseline to week 76 by BMI


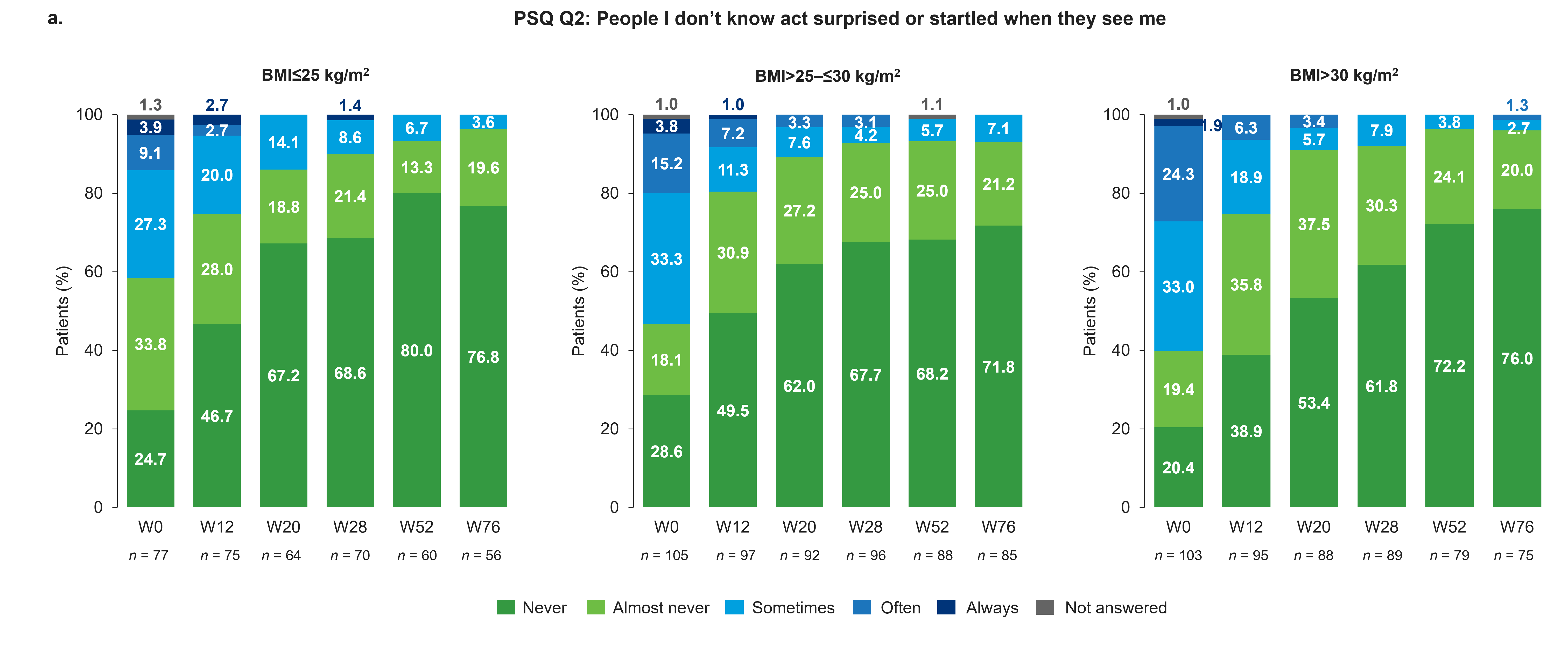


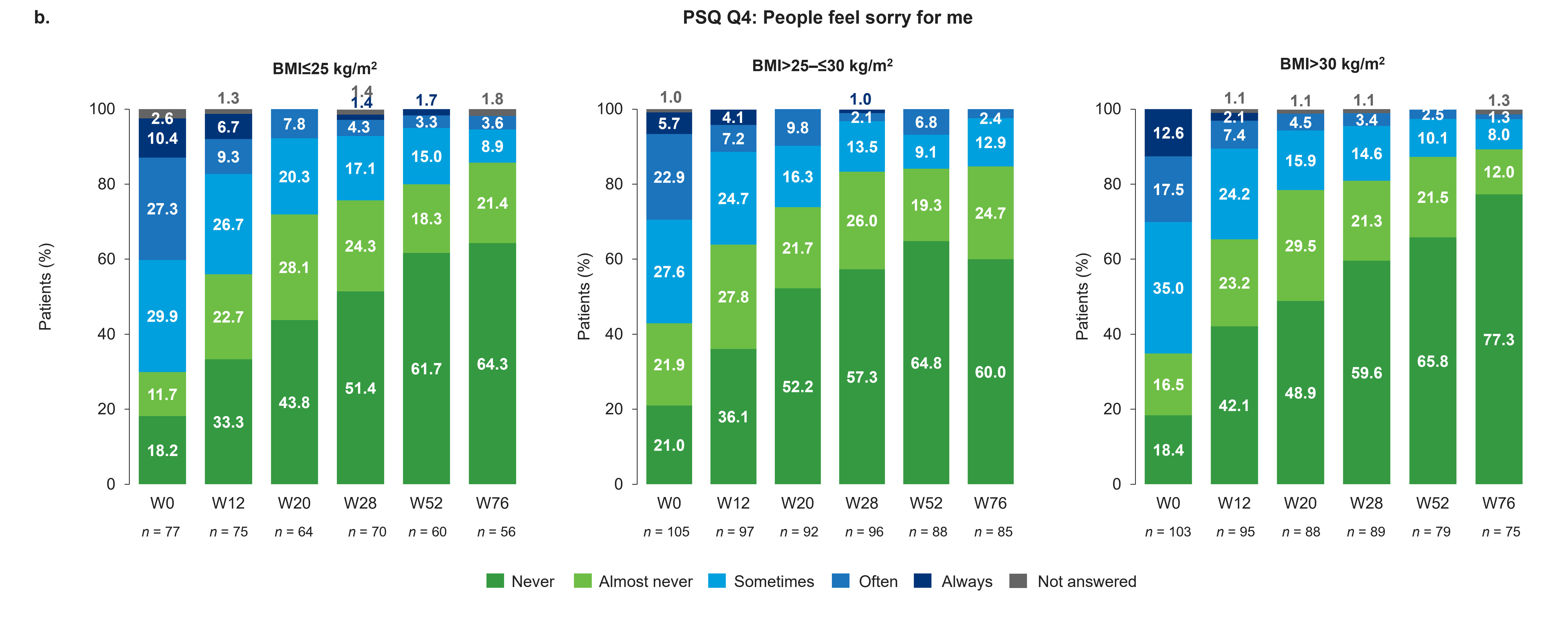


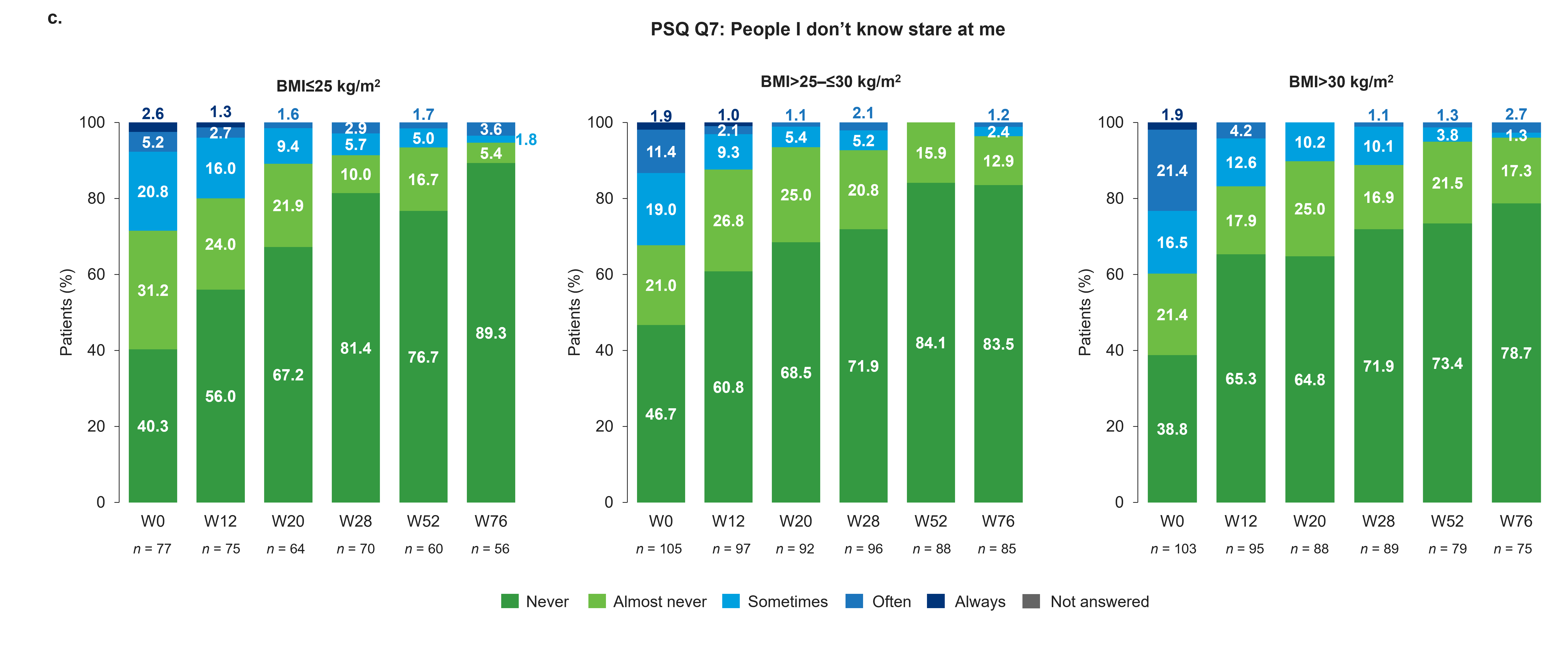


PSQ (a) Q2, (b) Q4, and (c) Q7 responses from baseline to W76 in subgroups of patients defined
by BMI.

BMI, body mass index; PSQ, Perceived Stigmatization Questionnaire; Q, Question; W, week.

**Figure S18** PSQ Q2, Q4, and Q7 responses from baseline to week 76 by depression at baseline


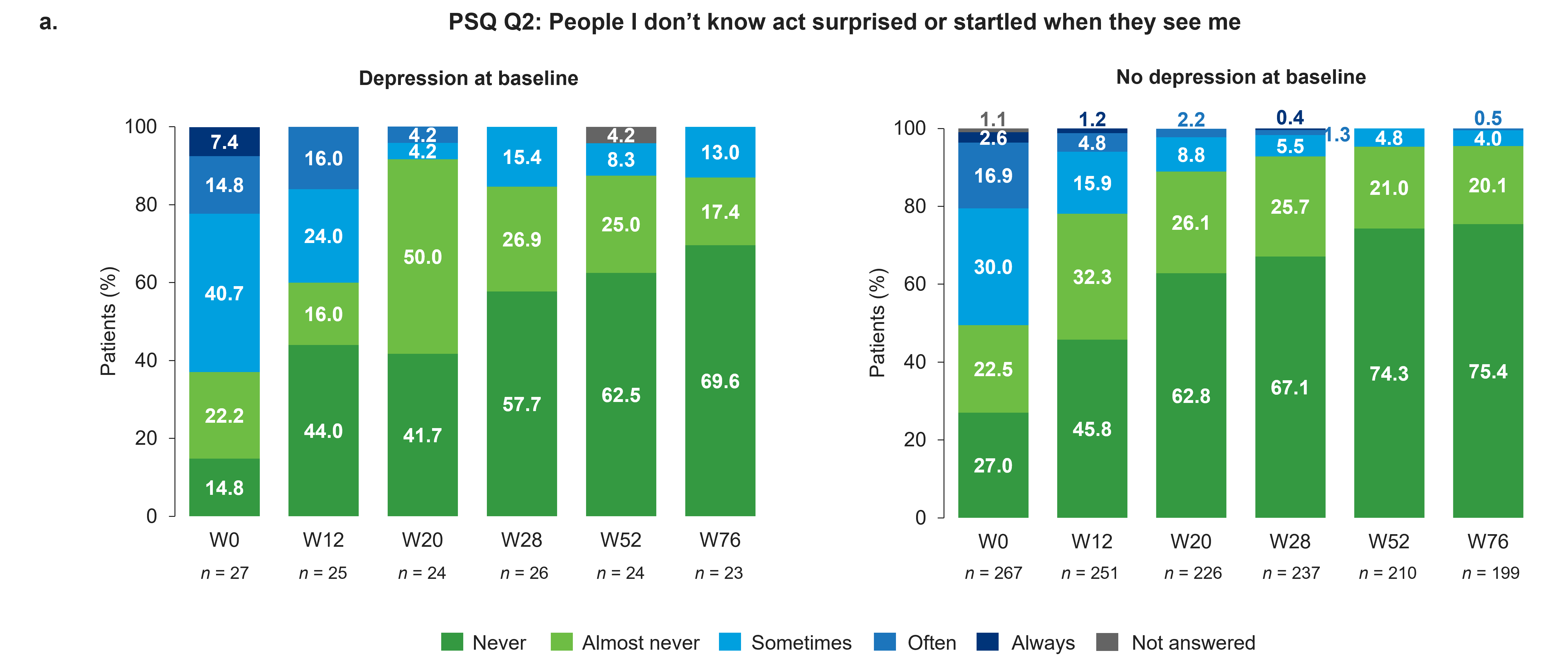


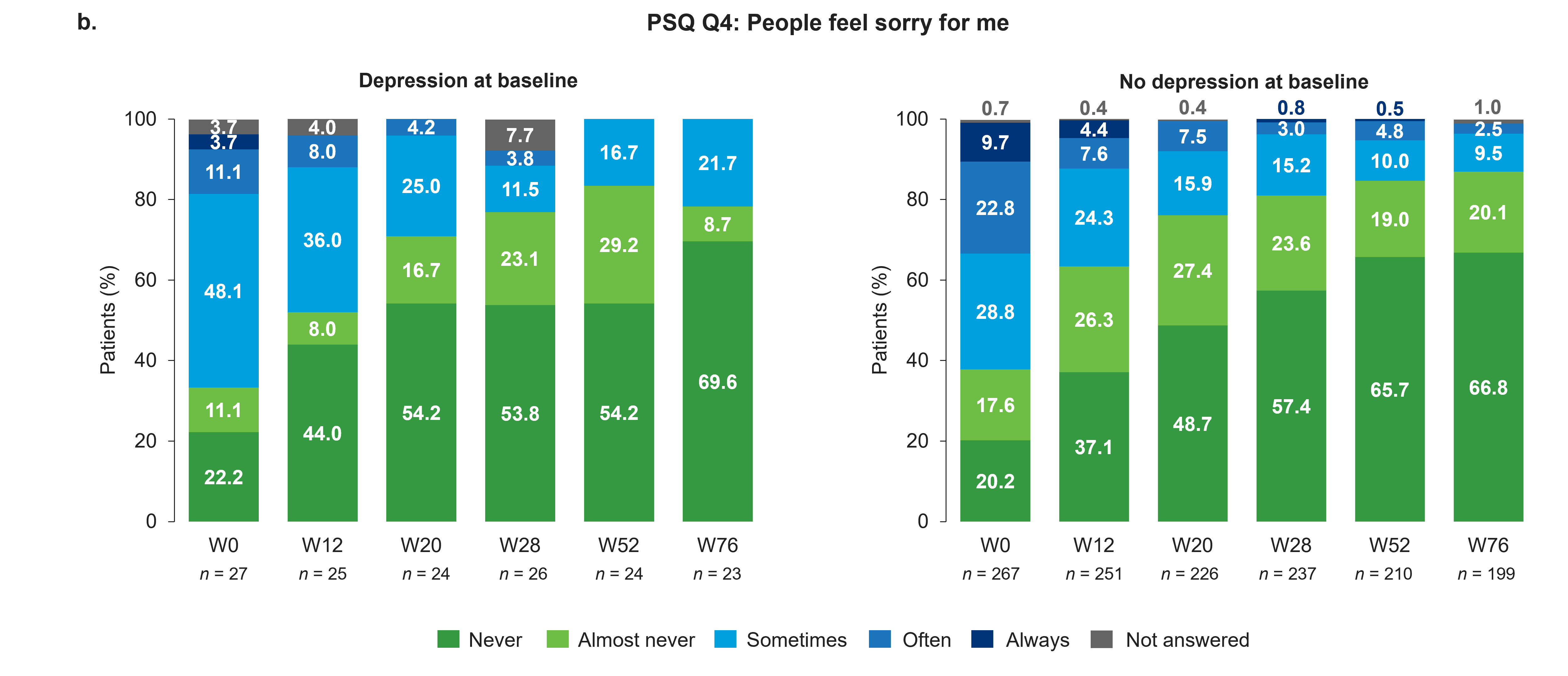


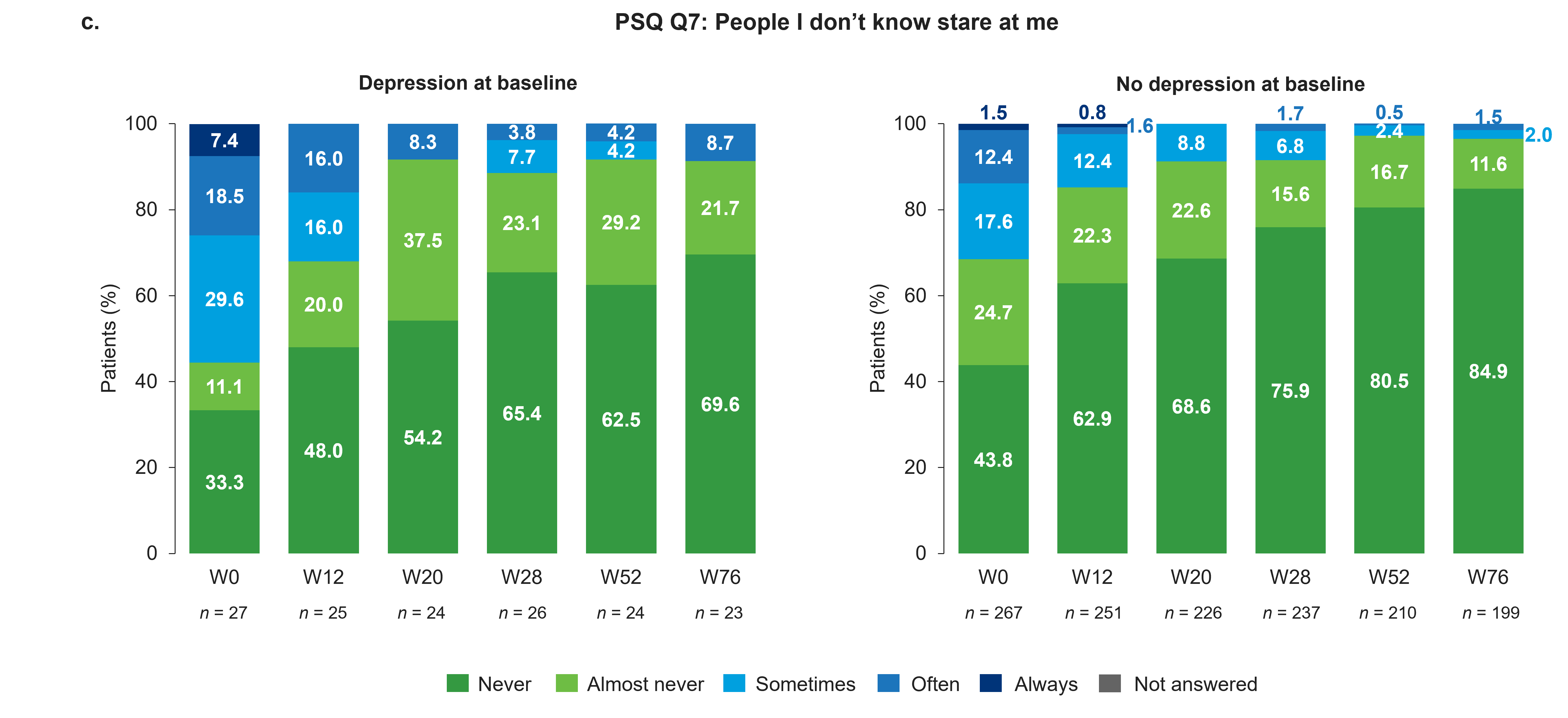


PSQ (a) Q2, (b) Q4, and (c) Q7 responses from baseline to W76 in subgroups of patients defined by presence or absence of depression at baseline.

PSQ, Perceived Stigmatization Questionnaire; Q, Question; W, week.

**Figure S19** PSQ Q2, Q4, and Q7 responses from baseline to week 76 by disease duration


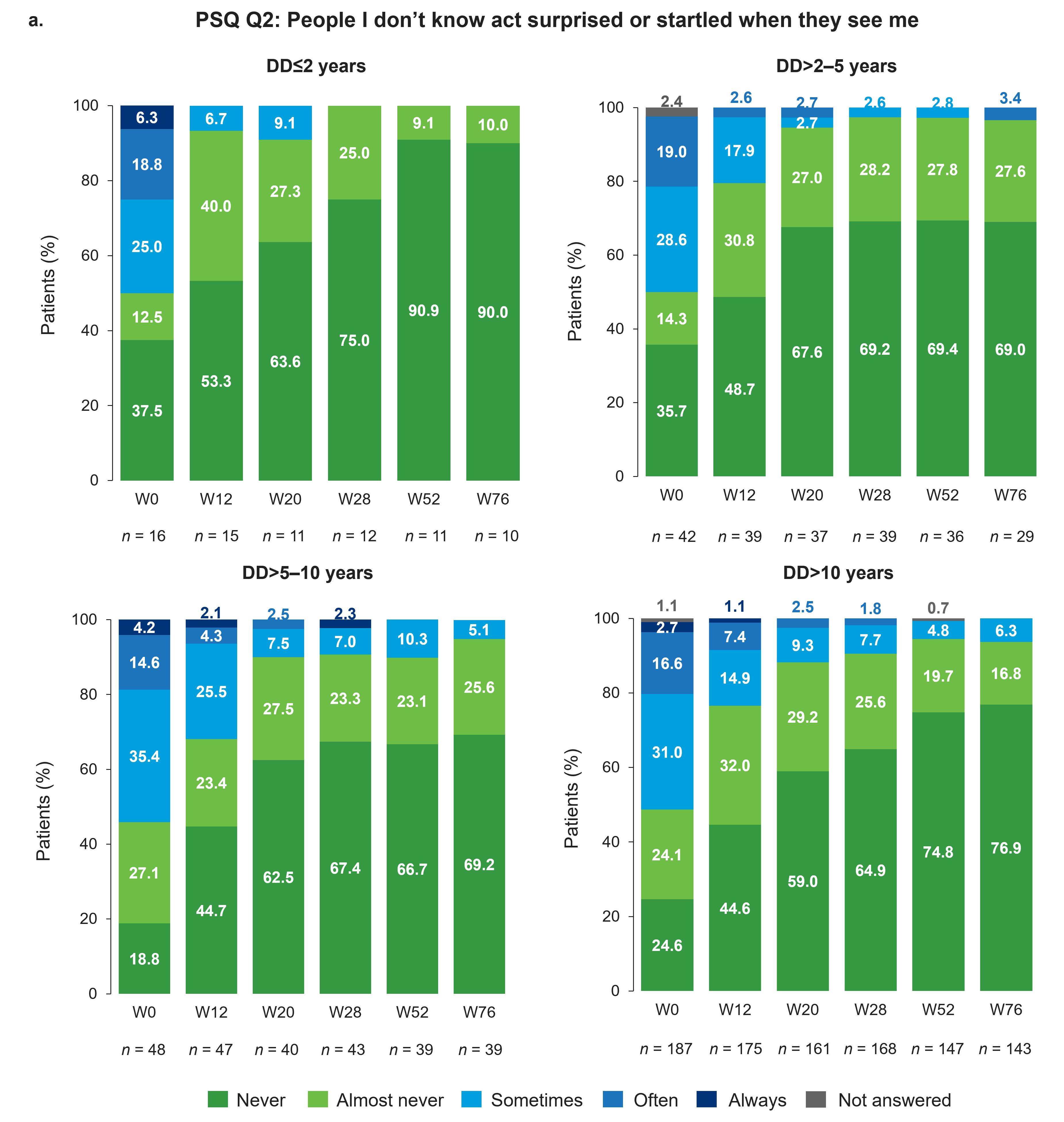


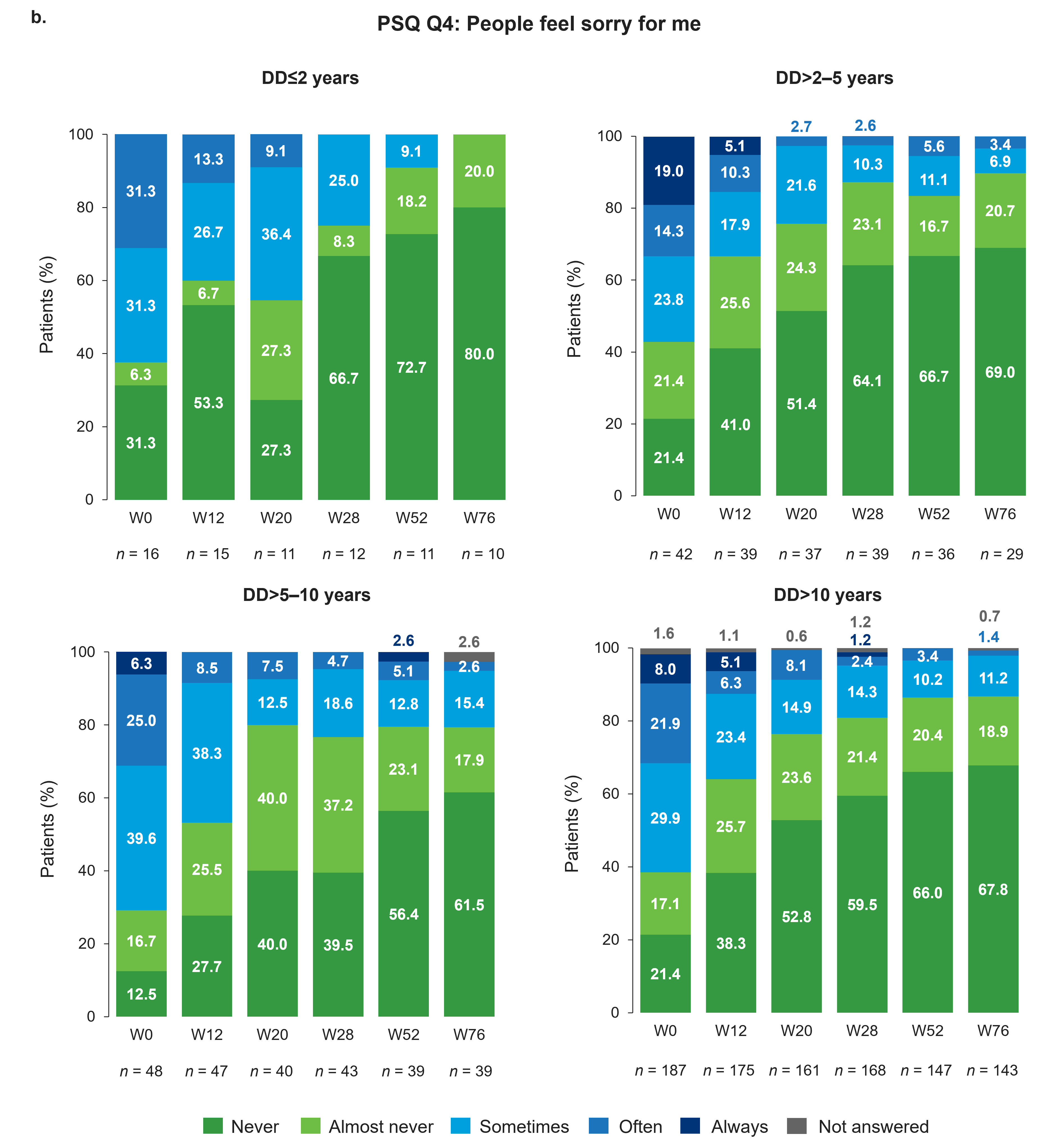


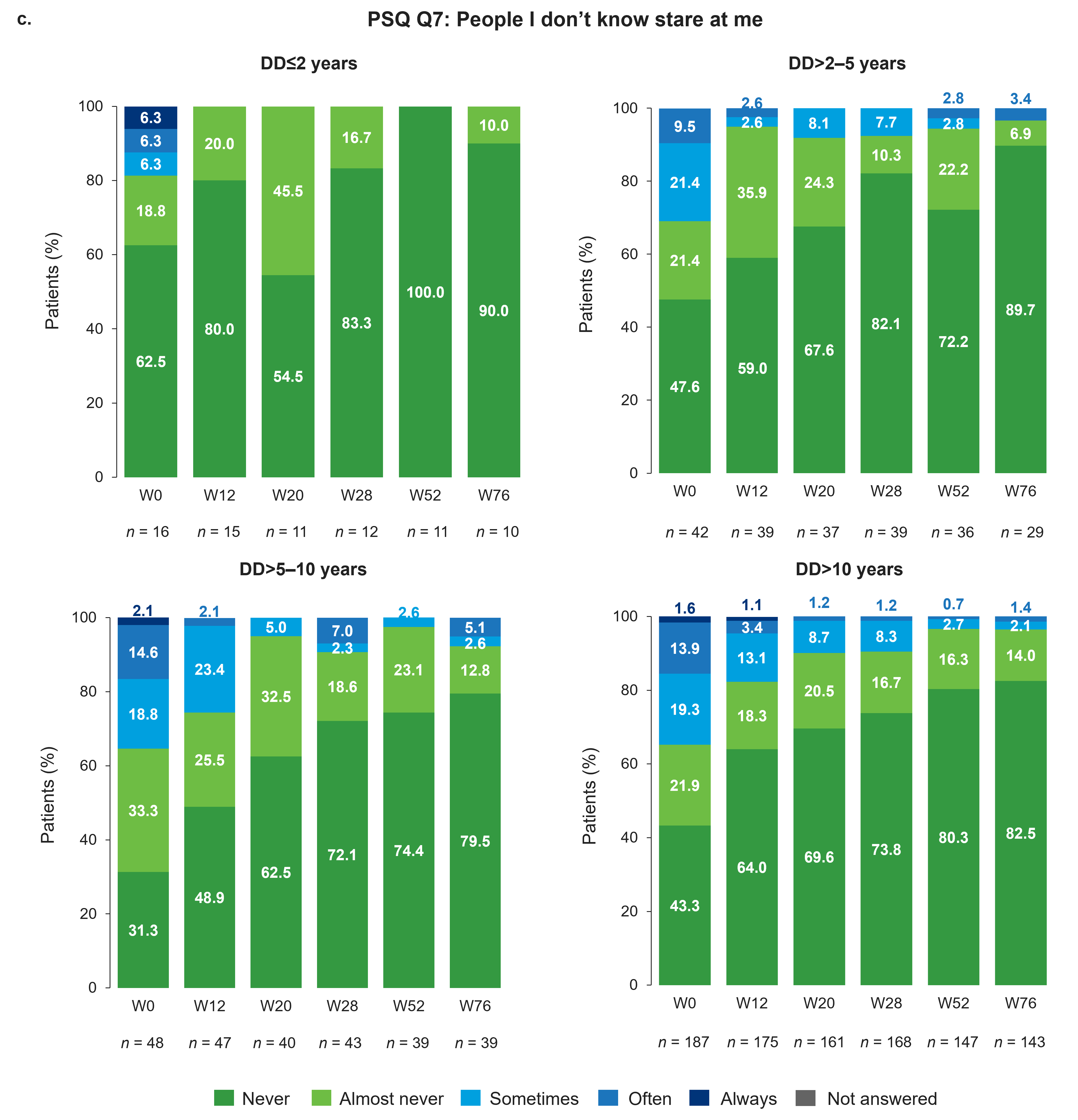


PSQ (a) Q2, (b) Q4, and (c) Q7 responses from baseline to W76 in subgroups of patients defined by disease duration.

DD, disease duration; PSQ, Perceived Stigmatization Questionnaire; Q, Question; W, week.

# References

1 Schmitt J , Wozel G. The psoriasis area and severity index is the adequate criterion to define severity in chronic plaque-type psoriasis. *Dermatology* 2005; **210**:194–9.

2 Spuls PI, Lecluse LL, Poulsen ML *et al.* How good are clinical severity and outcome measures for psoriasis? Quantitative evaluation in a systematic review. *J Invest Dermatol* 2010; **130**:933–43.

3 Langley RG, Feldman SR, Nyirady J *et al.* The 5-point Investigator's Global Assessment (IGA) Scale: a modified tool for evaluating plaque psoriasis severity in clinical trials. *J Dermatolog Treat* 2015; **26**:23–31.

4 Rich P , Scher RK. Nail Psoriasis Severity Index: a useful tool for evaluation of nail psoriasis. *J Am Acad Dermatol* 2003; **49**:206–12.

5 Davison NJ, Thompson AJ, Turner AJ *et al.* Generating EQ-5D-3L utility scores from the Dermatology Life Quality Index: a mapping study in patients with psoriasis. *Value Health* 2018; **21**:1010–8.

6 Finlay AY , Khan GK. Dermatology Life Quality Index (DLQI) – a simple practical measure for routine clinical use. *Clin Exp Dermatol* 1994; **19**:210–6.

7 Shikiar R, Willian MK, Okun MM *et al.* The validity and responsiveness of three quality of life measures in the assessment of psoriasis patients: results of a Phase II study. *Health Qual Life Outcomes* 2006; **4**:71.

8 Ryan C, Sadlier M, De Vol E *et al.* Genital psoriasis is associated with significant impairment in quality of life and sexual functioning. *J Am Acad Dermatol* 2015; **72**:978–83.

9 Lawrence JW, Fauerbach JA, Heinberg LJ *et al.* The reliability and validity of the Perceived Stigmatization Questionnaire (PSQ) and the Social Comfort Questionnaire (SCQ) among an adult burn survivor sample. *Psychol Assess* 2006; **18**:106–11.

10 Muller A, Smits D, Claes L *et al.* Validation of the German version of the Perceived Stigmatization Questionnaire/Social Comfort Questionnaire in adult burn survivors. *Burns* 2016; **42**:790–6.

11 Augustin M, Radtke MA, Zschocke I *et al.* The Patient Benefit Index: a novel approach in patient-defined outcomes measurement for skin diseases. *Arch Dermatol Res* 2009; **301**:561–71.

12 Feuerhahn J, Blome C, Radtke M *et al.* Validation of the patient benefit index for the assessment of patient-relevant benefit in the treatment of psoriasis. *Arch Dermatol Res* 2012; **304**:433-41.
